# Supplementary material for: Observation of coordinated RNA folding events by systematic cotranscriptional RNA structure probing
Source: Nat Commun. 2023 Nov 29;14:7839. doi: 10.1038/s41467-023-43395-9 (PMC10687018; doi:10.1038/s41467-023-43395-9)
Supplement: Supplementary file 1 — Supplementary Information [file 41467_2023_43395_MOESM1_ESM.pdf]

Observation of coordinated RNA folding events by systematic cotranscriptional RNA structure probing

Courtney E. Szyjka[1] and Eric J. Strobel[1*]

[1]Department of Biological Sciences, The University at Buffalo, Buffalo, NY 14260, USA

*To whom correspondence should be addressed: estrobel@buffalo.edu

This PDF file includes:

Supplementary Figures 1-24

Supplementary Tables 1-5

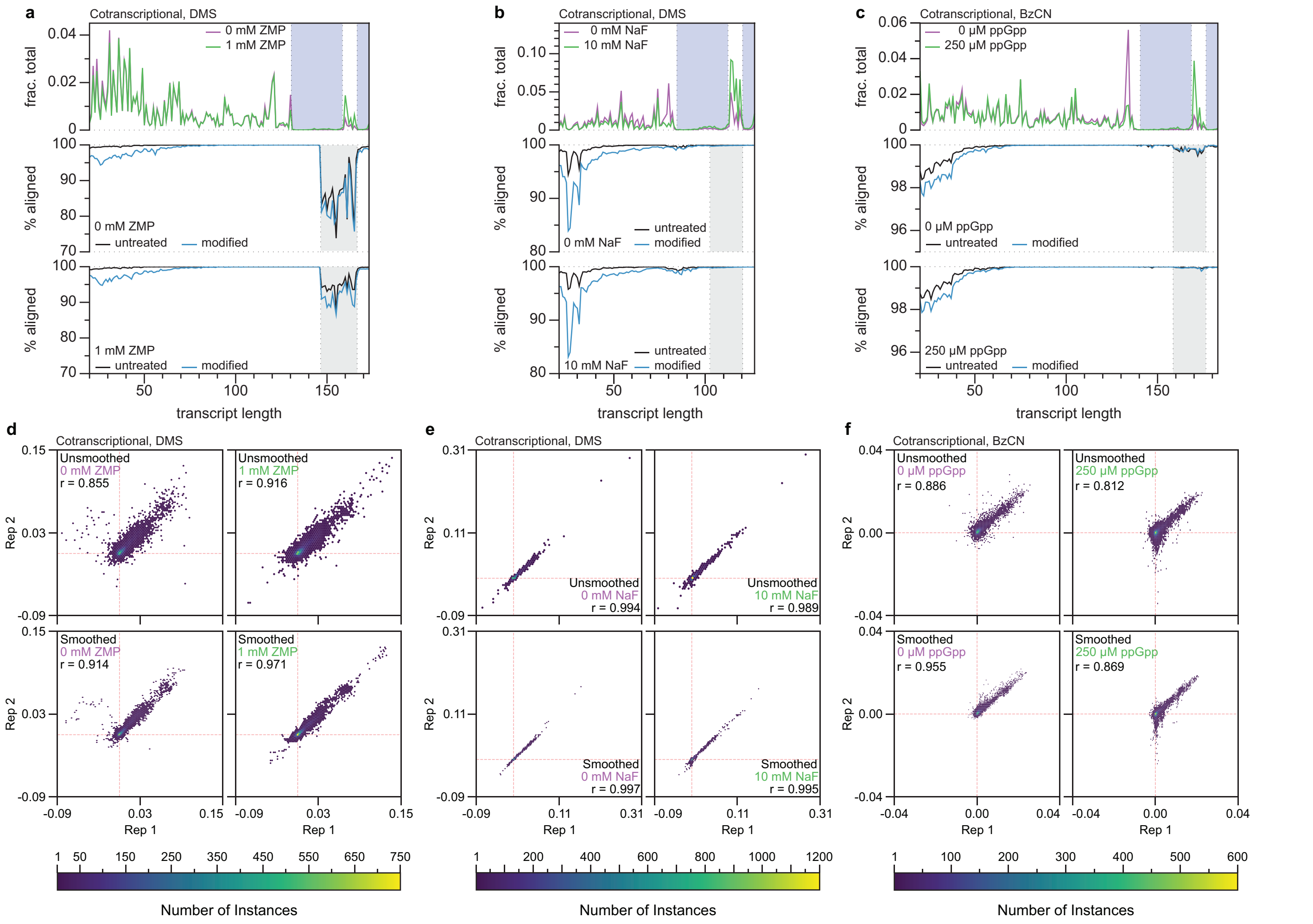

Supplementary Figure 1. TECprobe-VL performance benchmarks.
a-c, Plots showing the fraction of aligned reads that mapped to each transcript length (top) and the percentage of the reads kept after splitting those that aligned to each transcript length (middle, bottom) for the *Cbe pfl* ZTP riboswitch (DMS) (**a**), the *Bce crcB* fluoride riboswitch (DMS) (**b**), and the *Cba* ppGpp riboswitch (BzCN) (**c**) datasets. In the top plots, data from minus and plus ligand samples are purple and green, respectively. The blue shading in the top plot indicates transcripts that were not enriched by biotin-streptavidin roadblocking. The grey shading in the middle and lower plots indicates transcripts in which alignment was lower due to the presence of a nucleic acid species that is kept during fastq splitting but does not align. **d-f**, Hexbin plots comparing the reactivity of replicates for the *Cbe pfl* ZTP (DMS) (**d**), *Bce crcB* fluoride (DMS) (**e**), and *Cba* ppGpp (BzCN) (**f**) riboswitches for both unsmoothed (top) and smoothed (bottom) datasets. Data were plotted with a grid size of 75 by 75 hexagons, and the depth of overlapping data points is indicated by the heatmap. Red dashed lines indicate the position of 0 for each axis. BzCN, benzoyl cyanide; DMS, dimethyl sulfate. Source data are provided as a Source Data file.

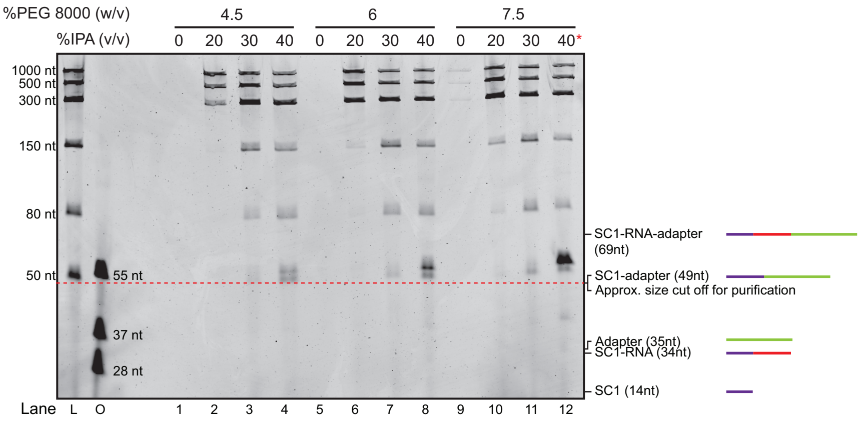

Supplementary Figure 2. Purification of single stranded nucleic acids using SPRI beads.
Denaturing PAGE analysis of nucleic acids purified using SPRI beads. Lanes L and O contain the Low Range ssRNA Ladder (New England Biolabs) and ssDNA oligonucleotide standards, respectively. The red asterisk marks the lane with the conditions used to purify RNA after the 3' adapter ligation in TECprobe-VL experiments. Size diagrams of some RNAs of interest present in TECprobe-VL experiments are shown on the right. The 5' SC1 hairpin (14nt - purple) is the smallest RNA before target RNA nucleotides are transcribed by RNAP. The first 19 target RNA transcript lengths are not analyzed in cotranscriptional structure probing experiments. Therefore, the SC1-RNA (34nt – purple and red) is the shortest RNA of interest and is a similar size to the adapter (35nt – green) before the RNA 3' adapter ligation. Using the conditions in lane 12, the size cut off for fragment retention is approximately 50nt, which is similar to the combined length of the ligated 5' SC1 hairpin and 3' adapter (49nt – purple and green). The selected conditions retain most nucleic acids >50nt, which includes the smallest TECprobe-VL ligation product of interest (SC1-RNA-adapter 69nt – purple, red, and green) and deplete most smaller fragments. The gel image is from n=1 experiment that was performed following seven experiments to identify optimal conditions. An uncropped gel image is provided in Supplementary Figure 24. PEG, polyethylene glycol; IPA, isopropyl alcohol; SC1, structure cassette 1.

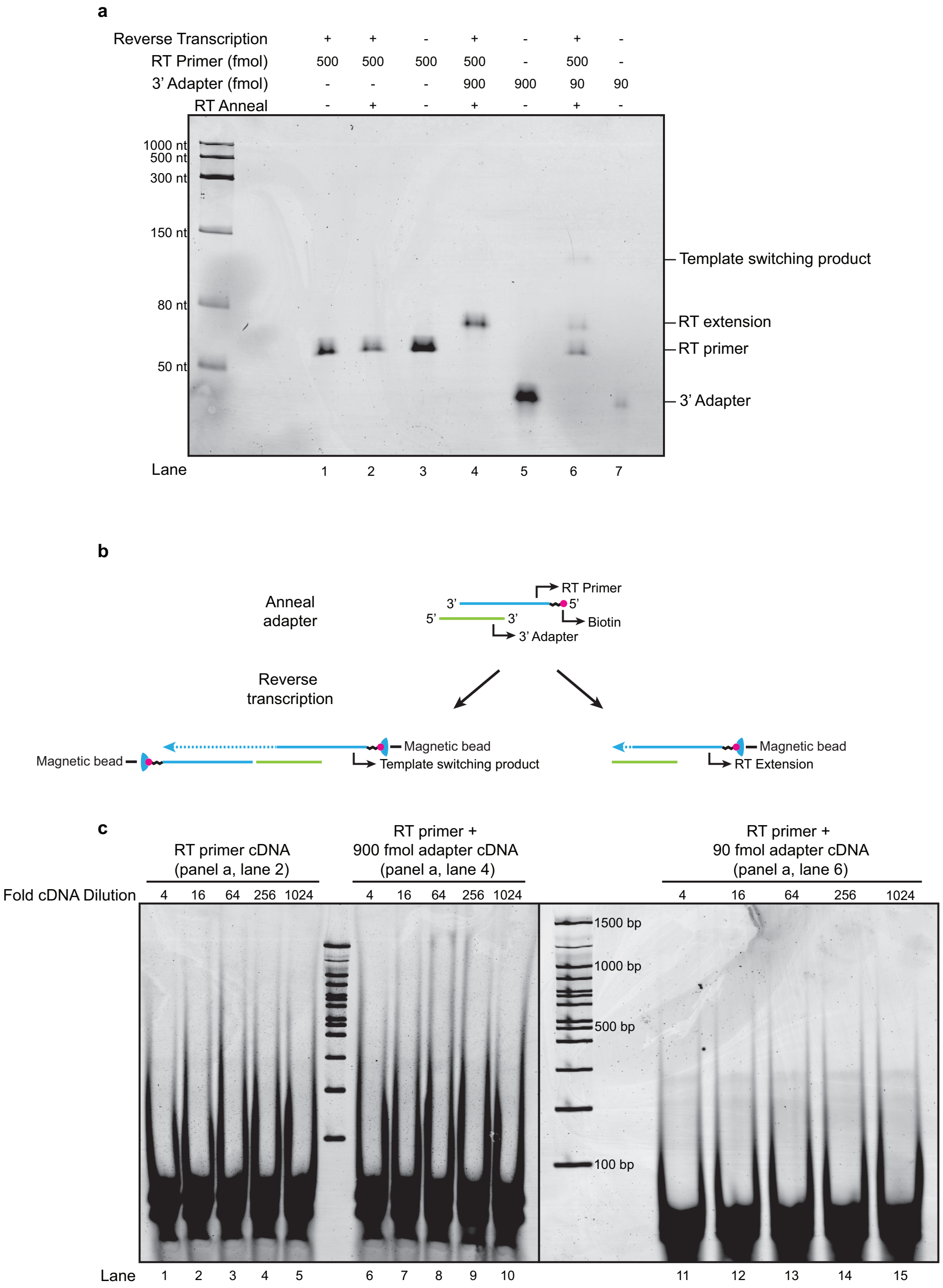

Supplementary Figure 3. Characterization of reverse transcription primer dimer products that result from template switching.
a, Denaturing PAGE analysis of reverse transcription reactions containing RT primer in the presence or absence of 3' adapter. Lanes 3, 5 and 7 contain input nucleic acids for size comparison and were not subjected to reverse transcription. 'RT anneal' indicates whether the RT anneal protocol was used to denature nucleic acids and anneal the RT primer before reverse transcription. **b**, Illustration of the RT primer template switching and extension products. **c**, Native PAGE analysis of PCR amplification products using cDNA from lanes 2, 4, and 6 in Supplementary Figure 3a as templates. Samples were diluted as indicated and subjected to 21 cycles of PCR amplification. The gel images are from n=1 experiment that agrees with the repeated observation of the unamplifiable template switching product during protocol optimization (e.g. Supplementary Figure 4a, c). Uncropped gel images are provided in Supplementary Fig. 24. RT, reverse transcription.

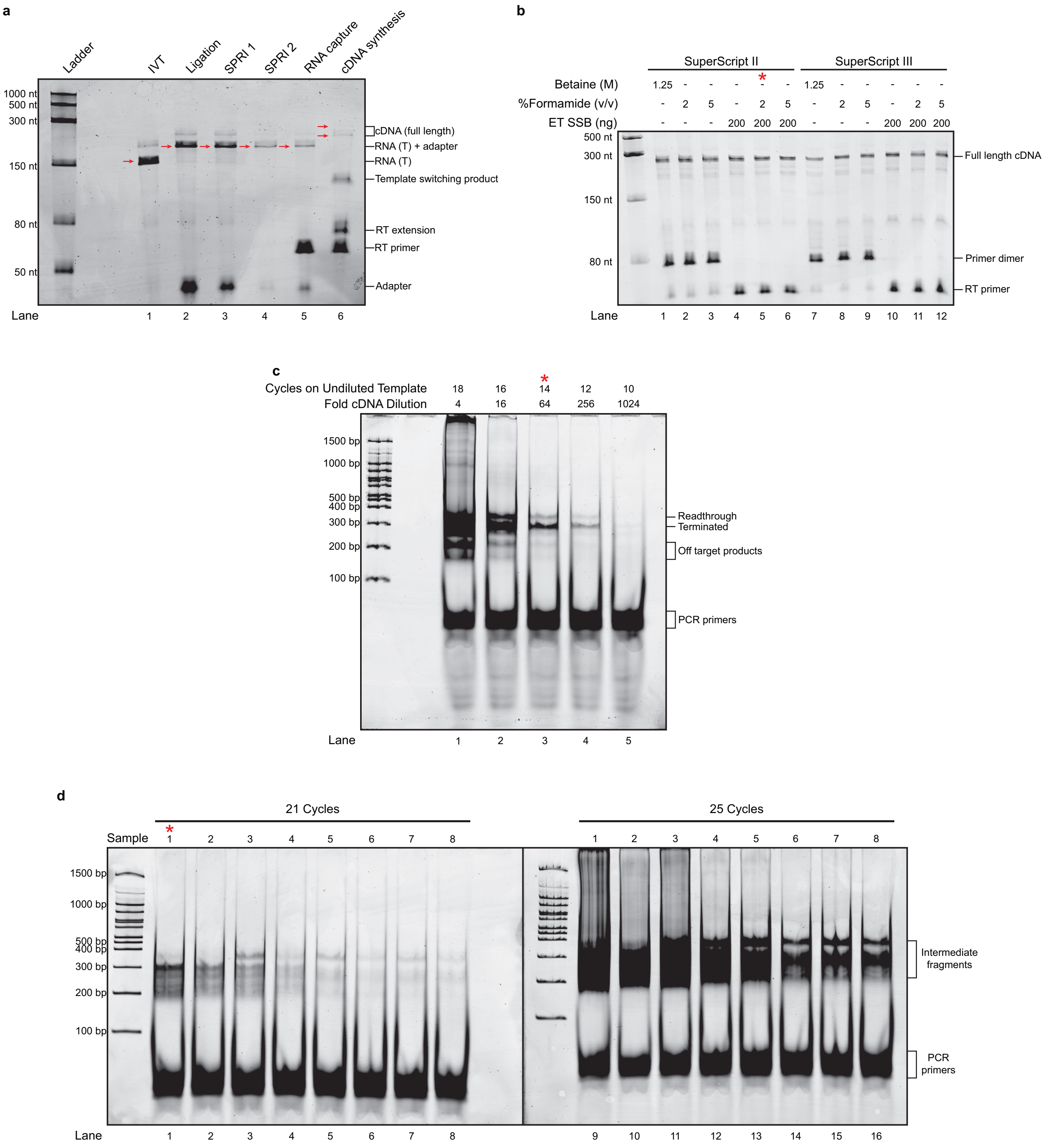

Supplementary Figure 4. TECprobe RNA processing and cDNA amplification.
a, Denaturing PAGE analysis of TECprobe-VL processing intermediates. *Cba* ppGpp riboswitch DNA templates without randomly biotinylated nucleotides were used to produce two prominent bands for easier visualization of sample processing. The identities of bands indicated by red arrows are shown on the right. **b**, Denaturing PAGE analysis of reverse transcription reactions using SuperScript II or III reverse transcriptase and various combinations of additives. A *pfl* ZTP riboswitch template containing a biotin-TEG stall site and inactivated transcription terminator was used. The red asterisk represents the conditions used for TECprobe experiments. **c**, Test amplification (21 cycles) using serial dilutions of the cDNA from panel a, lane 6. Readthrough and terminated products represent the PCR products of cDNA that was produced from

readthrough and terminated transcripts, respectively. Fold cDNA dilution indicates the dilution of the cDNA, relative to the undiluted cDNA, that was subjected to PCR. Cycles on undiluted template indicates the number of cycles needed to achieve the same concentration of PCR product using 12 μl of undiluted cDNA. The red asterisk represents the lane with the appropriate band intensity to target when amplifying cDNA from TECprobe-SL experiments. **d**, Native PAGE analysis of dsDNA libraries generated using 1:16 diluted cDNA from TECprobe-VL experiments targeting the *Cba* ppGpp riboswitch after 21 cycles (left) or 25 cycles (right) of PCR. The red asterisk indicates the appropriate band intensity to target when amplifying cDNA from TECprobe-VL experiments. The gel images in panels a-c are representative of at least n=2 replicates. The gel images in panels d and e are representative of test amplifications that were performed for every TECprobe library in this work. Uncropped gel images are provided in Supplementary Fig. 24. IVT, *in vitro* transcription; SPRI, solid-phase reversible immobilization; T, terminated; RT, reverse transcription; ET SSB, extreme thermostable single-stranded DNA binding protein; TEG, triethylene glycol.

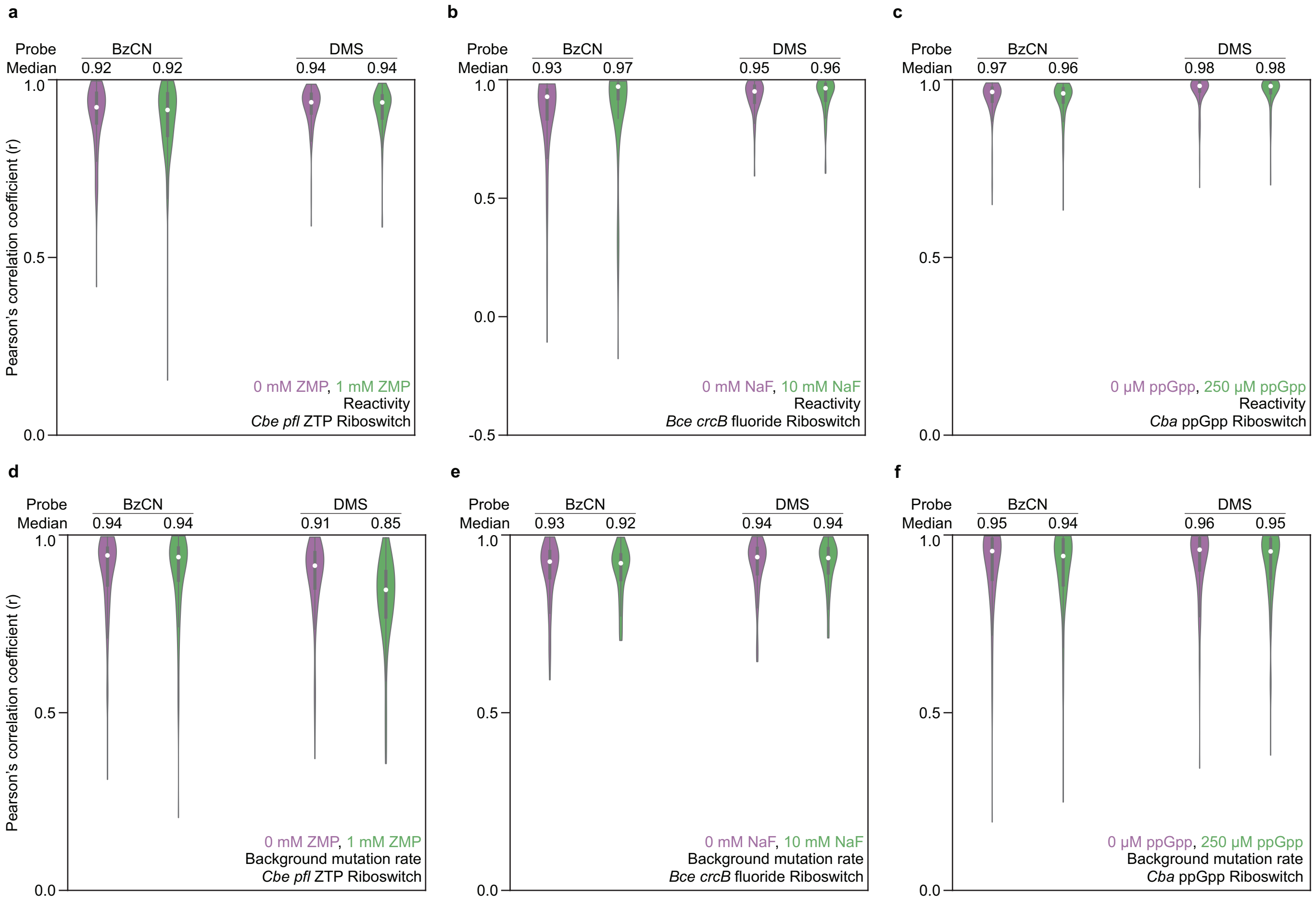

Supplementary Figure 5. Comparison of reactivity and background mutation rates for neighboring transcripts.
Violin plots of Pearson's correlation coefficients comparing reactivities (**a-c**) or background mutation rates (**d-f**) of neighboring transcripts (*n* and *n*+1) for the *Cbe pfl* ZTP (**a** and **d**), *Bce crcB* fluoride (**b** and **e**), and *Cba* ppGpp (**c** and **f**) riboswitches. White circles indicate the data median. Source data are provided as a Source Data file. BzCN, benzoyl cyanide; DMS, dimethyl sulfate.

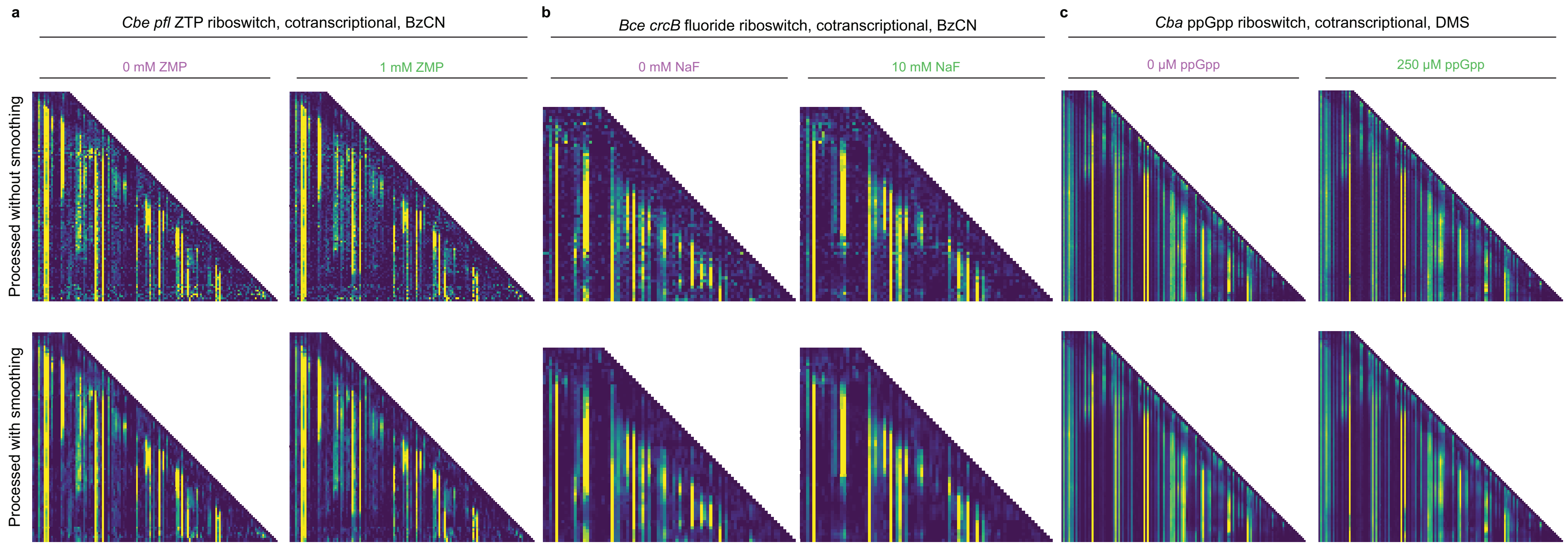

Supplementary Figure 6. Comparison of reactivity matrices with and without neighboring transcript smoothing. TECprobe-VL reactivity matrices for the *Cbe pfl* ZTP (BzCN) (**a**), *Bce crcB* fluoride (BzCN) (**b**), and *Cba* ppGpp (DMS) (**c**) riboswitches. Matrices were generated from data processed without (top) or with (bottom) neighboring transcript smoothing. Reactivity is shown as background-subtracted mutation rate. Source data are provided as a Source Data file. BzCN, benzoyl cyanide; DMS, dimethyl sulfate.

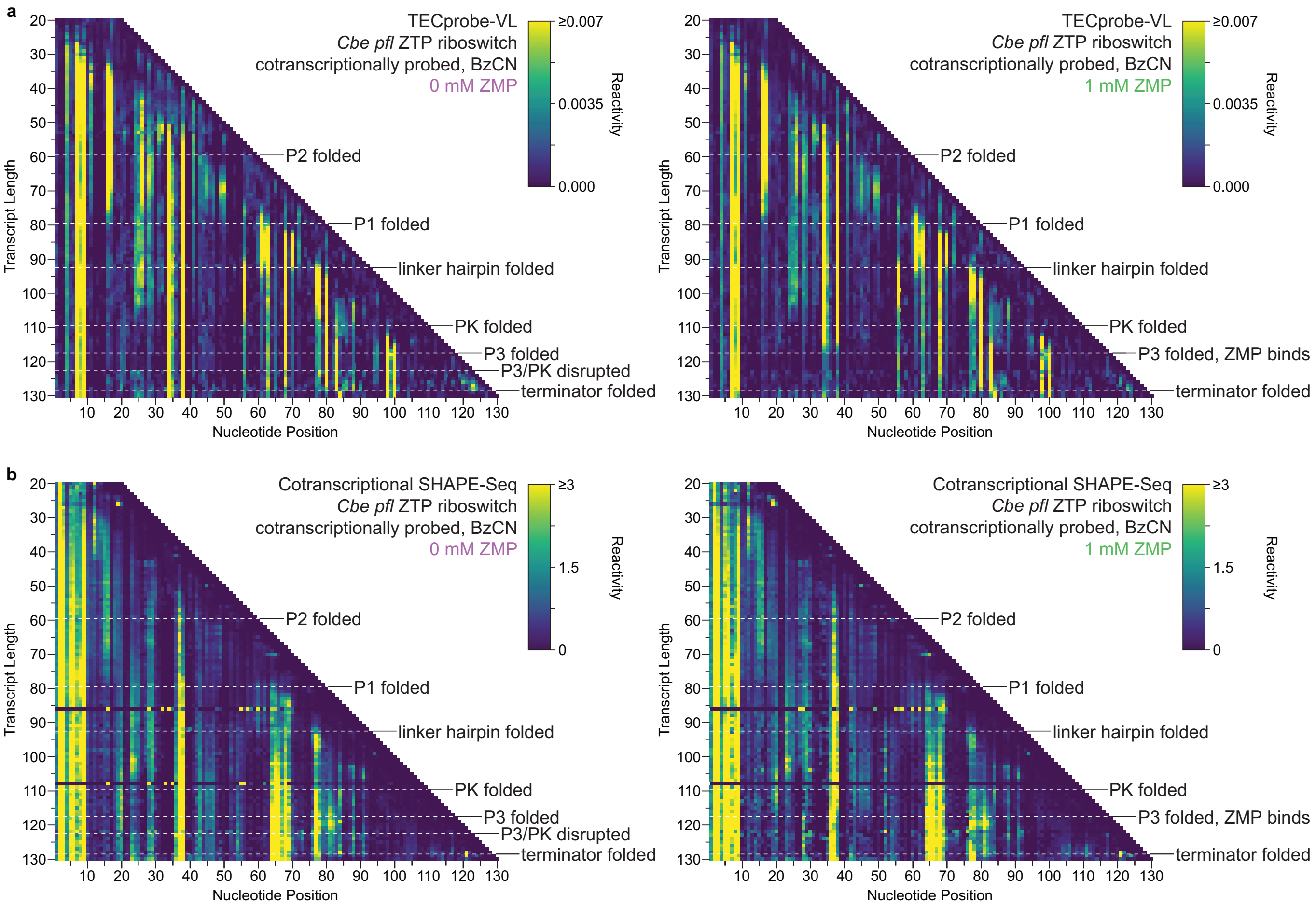

Supplementary Figure 7. Comparison of TECprobe-VL and Cotranscriptional SHAPE-Seq data: *C. beijerinckii pfl* ZTP riboswitch reactivity matrices.

a, TECprobe-VL BzCN reactivity matrices for the *pfl* ZTP riboswitch with 0 mM and 1 mM ZMP. Data are from Figure 3c, d and are presented here for comparison to cotranscriptional SHAPE-Seq data. **b**, Cotranscriptional SHAPE-Seq BzCN reactivity matrices for the *pfl* ZTP riboswitch with 0mM and 1mM ZMP. Data are from Strobel et al., 2019, *A ligand-gated strand displacement mechanism for ZTP riboswitch transcription control*, *Nat Chem Biol.* 2019 Nov; 15(11):1067-107 (Ref. 33). Source data are provided as a Source Data file. BzCN, benzoyl cyanide; PK, pseudoknot.

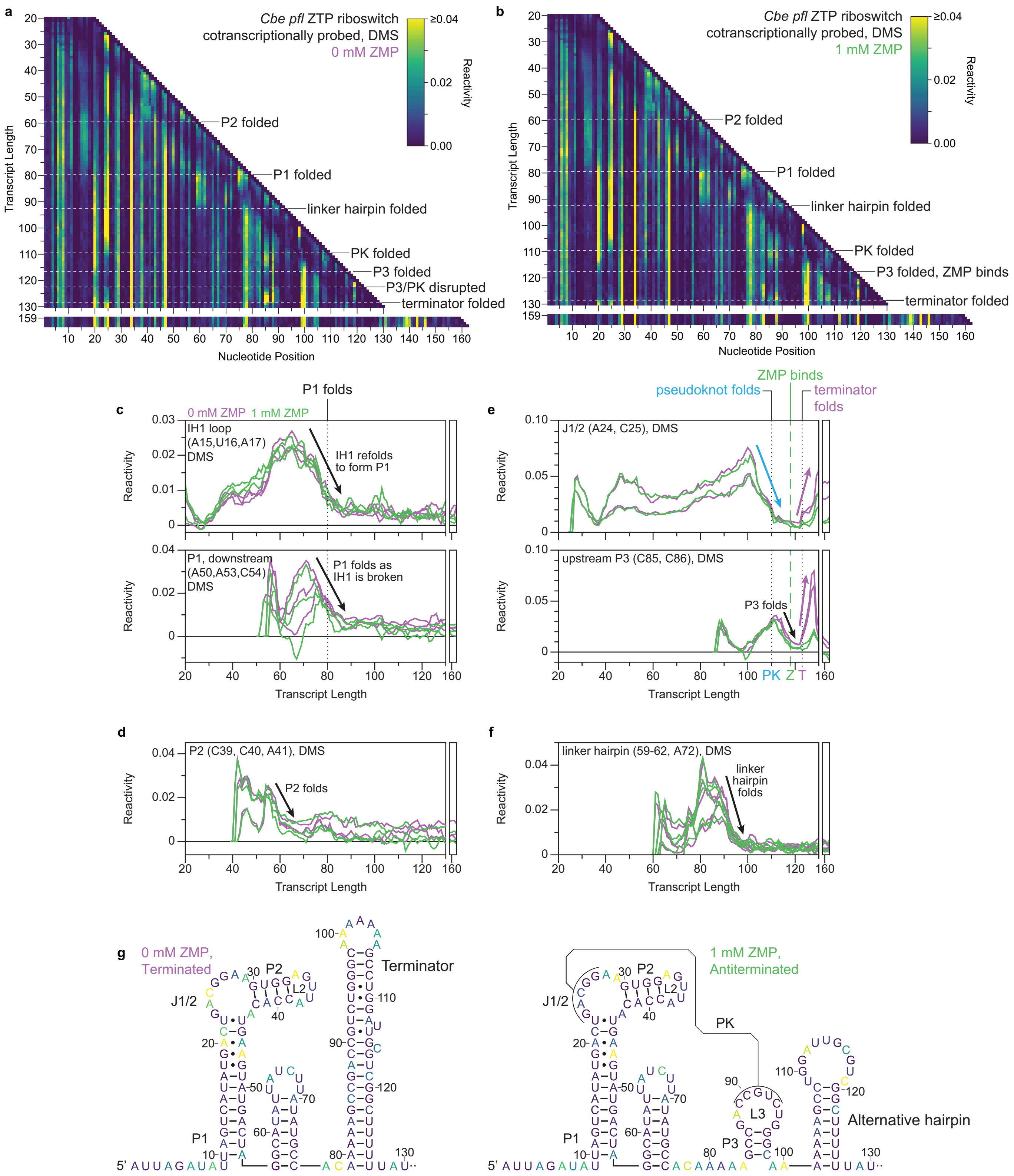

Supplementary Figure 8. Cotranscriptional DMS probing of the *C. beijerinckii pfl* ZTP riboswitch. **a**, **b**, TECprobe-VL DMS reactivity matrices for the *Cbe pfl* ZTP riboswitch with 0 mM and 1 mM ZMP. Transcripts 131-158, which were not enriched, are excluded. Reactivity is shown as background-subtracted mutation rate. Data are from two independent replicates that were concatenated and analyzed together. **c-f**, Transcript length-dependent reactivity changes showing the IH1-to-P1 (**c**), P2 formation (**d**), pseudoknot formation/disruption (**e**), and linker hairpin formation (**f**) folding transitions. Data from 0 mM and 1 mM ZMP samples are purple and green, respectively. Vertical dotted and dashed lines mark when the indicated folding

transitions occur. **g**, *pfl* ZTP riboswitch secondary structures colored by DMS reactivity (terminated: 130 nt, antiterminated: 160 nt). Source data are provided as a Source Data file. DMS, dimethyl sulfate; PK, pseudoknot.

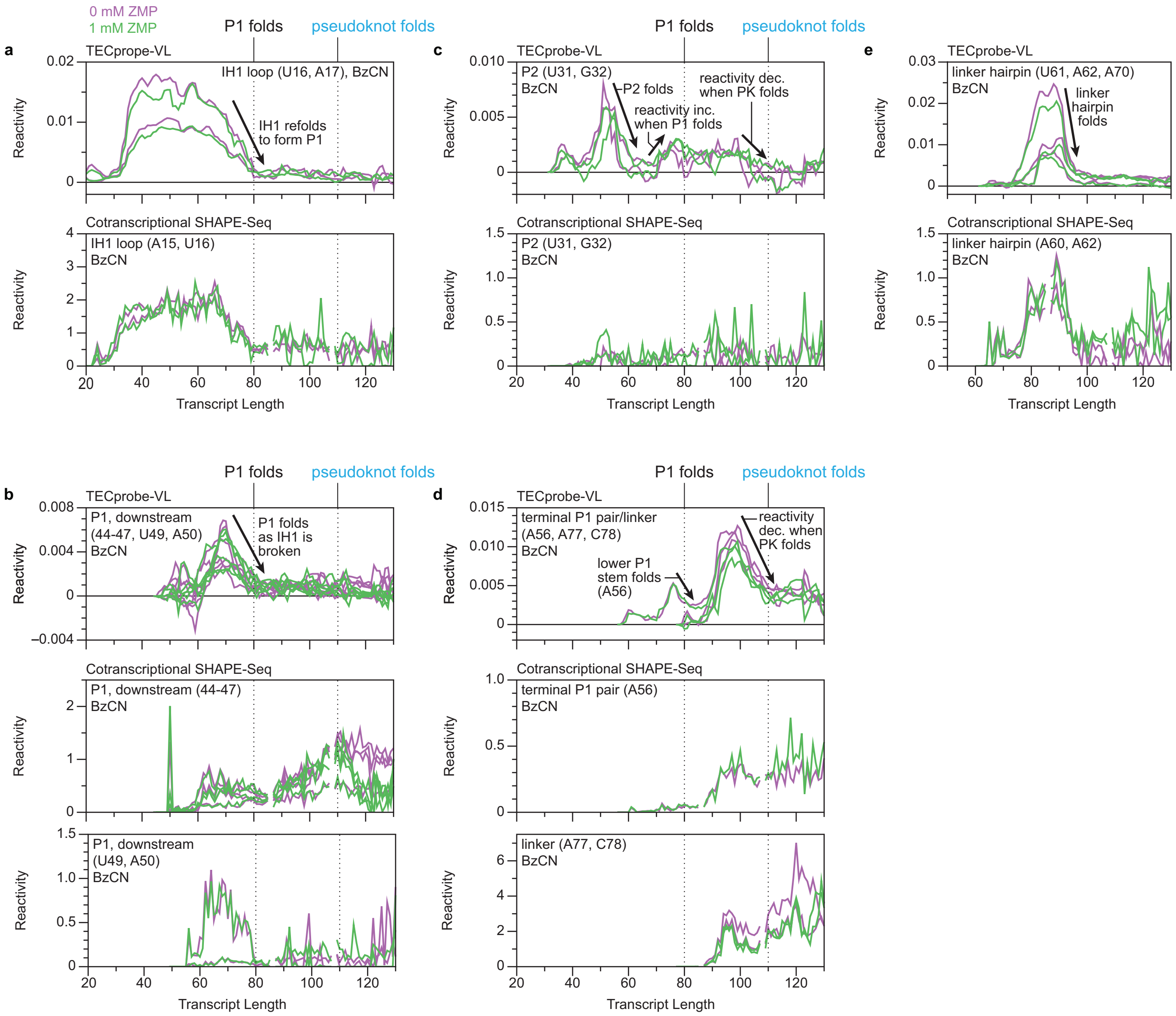

Supplementary Figure 9. Comparison of TECprobe-VL and Cotranscriptional SHAPE-Seq data: *C. beijerinckii pfl* ZTP riboswitch P1 stem and linker hairpin folding.

Plots of TECprobe-VL (top) and Cotranscriptional SHAPE-Seq (bottom) transcript length-dependent BzCN reactivity changes showing the IH1-to-P1 transition (**a, b**), P2 formation (**c**), linker stabilization (**d**), and linker hairpin formation (**e**). Data from 0 mM and 1 mM ZMP samples are purple and green, respectively. Vertical dotted lines mark when the indicated folding transitions occur. In panels b and d, reactivity trajectories that were plotted together for TECprobe-VL data are shown separately for Cotranscriptional SHAPE-Seq data to facilitate clear visualization of the data. TECprobe-VL data are from Figure 3c,d. Cotranscriptional SHAPE-Seq data are from Strobel et al., 2019, *A ligand-gated strand displacement mechanism for ZTP riboswitch transcription control*, *Nat Chem Biol.* 2019 Nov; 15(11):1067-1076 (Ref. 33). Source data are provided as a Source Data file. BzCN, benzoyl cyanide; PK, pseudoknot.

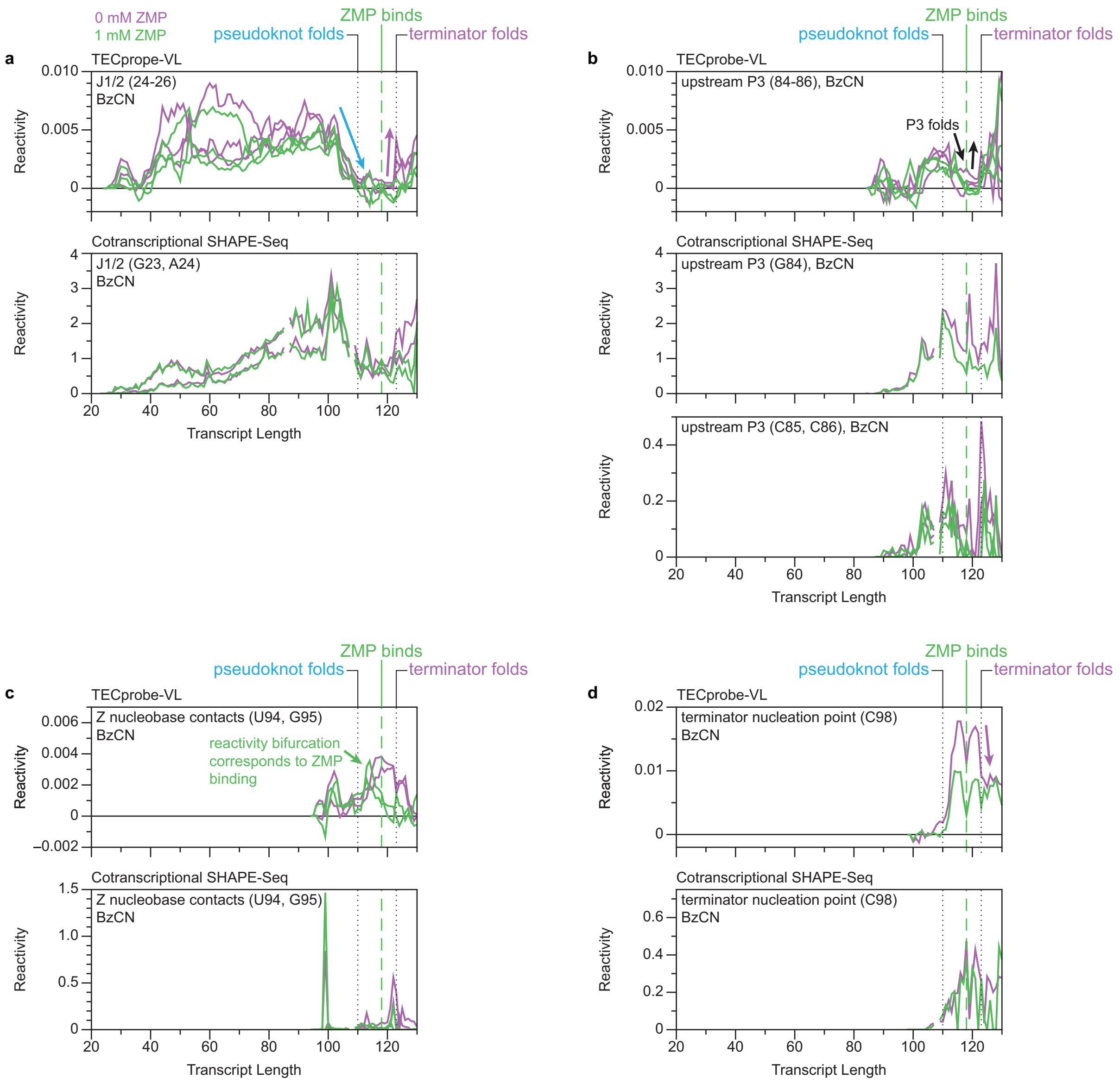

Supplementary Figure 10. Comparison of TECprobe-VL and Cotranscriptional SHAPE-Seq data: *C. beijerinckii pfl* ZTP riboswitch pseudoknot formation/disruption and P3 stem stabilization. Plots of TECprobe-VL (top) and Cotranscriptional SHAPE-Seq (bottom) transcript length-dependent BzCN reactivity changes showing pseudoknot formation/disruption (**a**), P3 formation (**b**), ZMP coordination (**c**), and P3 stabilization (**d**). Data from 0 mM and 1 mM ZMP samples are purple and green, respectively. In panel b, reactivity trajectories that were plotted together for TECprobe-VL data are shown separately for Cotranscriptional SHAPE-Seq data to facilitate clear visualization of the data. TECprobe-VL data are from Figure 3c,d. Cotranscriptional SHAPE-Seq data are from Strobel et al., 2019, *A ligand-gated strand displacement mechanism for ZTP riboswitch transcription control*, *Nat Chem Biol.* 2019 Nov; 15(11):1067-1076 (Ref. 33). Source data are provided as a Source Data file. BzCN, benzoyl cyanide.

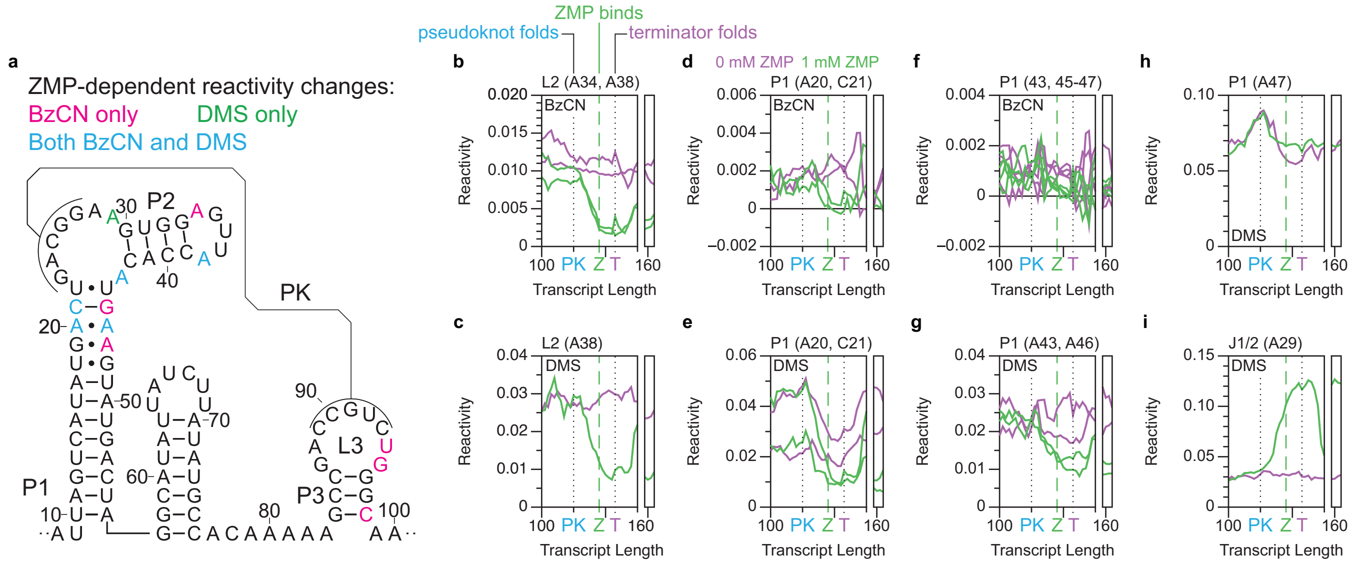

Supplementary Figure 11. ZMP-dependent reactivity changes in the *C. beijerinckii pfl* ZTP aptamer. **a**, Secondary structure of the *pfl* ZTP aptamer colored to show nucleotides that undergo ZMP-dependent reactivity changes observed using BzCN, DMS, or both probes. **b-i**, Plots showing ZMP-dependent reactivity changes in L2 (**b, c**), P1 (**d-g**), and J1/2 (**i**), and the absence of a ZMP-dependent change in the DMS reactivity of A47 (**h**). Data from 0 mM and 1 mM ZMP samples are purple and green, respectively. Vertical dotted and dashed lines mark when the indicated folding transitions occur. BzCN data are from Figure 3c, d. DMS data are from Supplementary Figure 8a, b. Source data are provided as a Source Data file. BzCN, benzoyl cyanide; DMS, dimethyl sulfate.

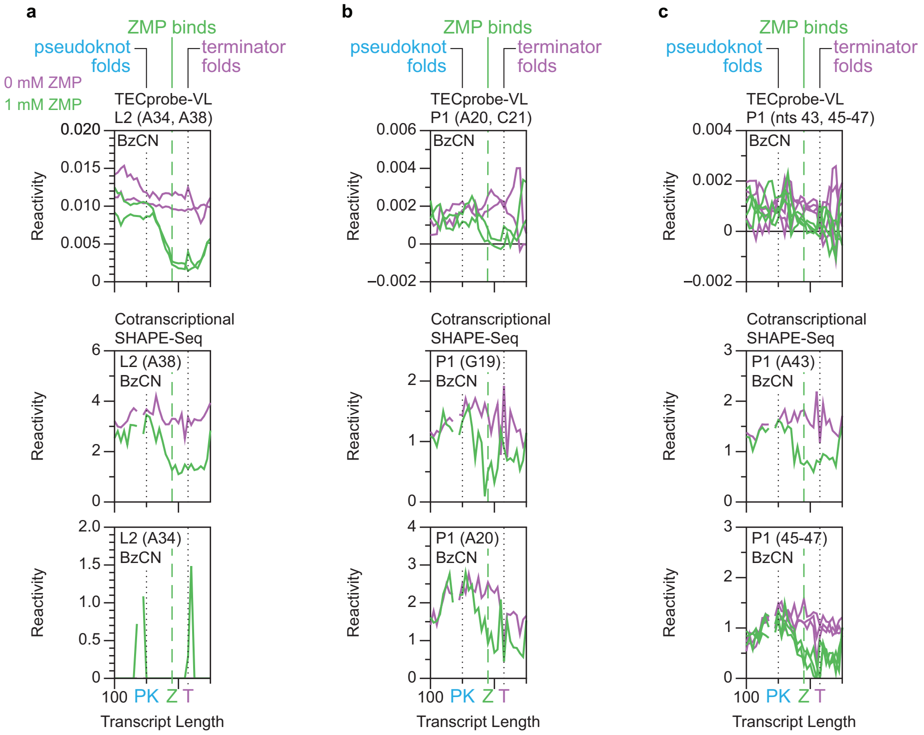

Supplementary Figure 12. Comparison of TECprobe-VL and Cotranscriptional SHAPE-Seq data: *C. beijerinckii pfl* ZTP riboswitch ZMP-dependent aptamer stabilization

Plots of TECprobe-VL (top) and Cotranscriptional SHAPE-Seq (bottom) transcript length-dependent BzCN reactivity changes showing the ZMP-dependent reactivity changes in L2 (**a**), upstream P1 (**b**), and downstream P1 (**c**). Data from 0 mM and 1 mM ZMP samples are purple and green, respectively. Reactivity trajectories that were plotted together for TECprobe-VL data are shown separately for Cotranscriptional SHAPE-Seq data to facilitate clear visualization of the data. TECprobe-VL data are from Figure 3c,d. Cotranscriptional SHAPE-Seq data are from Strobel et al., 2019, *A ligand-gated strand displacement mechanism for ZTP riboswitch transcription control*, *Nat Chem Biol.* 2019 Nov; 15(11):1067-1076 (Ref. 33). Source data are provided as a Source Data file. BzCN, benzoyl cyanide; PK, pseudoknot folds; Z, ZMP binds; T, terminator folds.

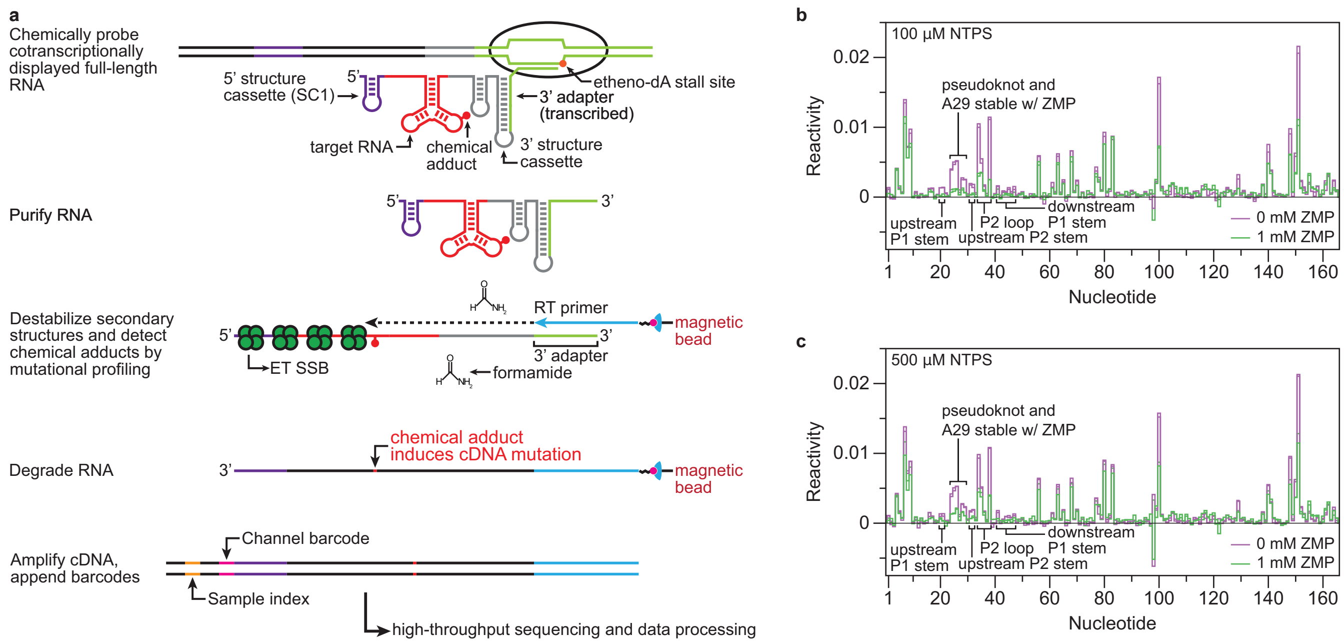

Supplementary Figure 13. Cotranscriptional probing of full-length *C. beijerinckii pfl* ZTP riboswitch transcripts using TECprobe-SL.
a, Illustration of the TECprobe-SL procedure. DNA templates that contain an internal etheno-dA modification are *in vitro* transcribed to generate roadblocked TECs that contain cotranscriptionally folded RNA. A 3' structure cassette is used to sequester the 3' adapter sequence from interactions that could influence target RNA structure. Cotranscriptionally displayed RNA is then chemically probed. The transcribed sequence contains the 3' adapter so no ligation is needed. Following cotranscriptional chemical probing, solid-phase error-prone reverse transcription is performed. Full-length cDNA is amplified using primers that anneal to the 3' adapter and the 5' structure cassette. The resulting libraries are sequenced and processed using fastp[78], TECtools, and ShapeMapper2[79]. **b**, **c** TECprobe-SL reactivity profiles for cotranscriptionally BzCN-probed full length *Cbe pfl* ZTP riboswitch transcripts with 100 µM (**b**) or 500 µM (**c**) NTPs. The transcription terminator was inactivated by mutations to the poly-U tract to promote full-length RNA synthesis in the absence of ZMP. Data from 0 mM and 1 mM ZMP samples are purple and green, respectively. Two independent replicates are plotted for each condition. Source data are provided as a Source Data file. RT, reverse transcription; ET SSB, extreme thermostable single-stranded DNA binding protein.

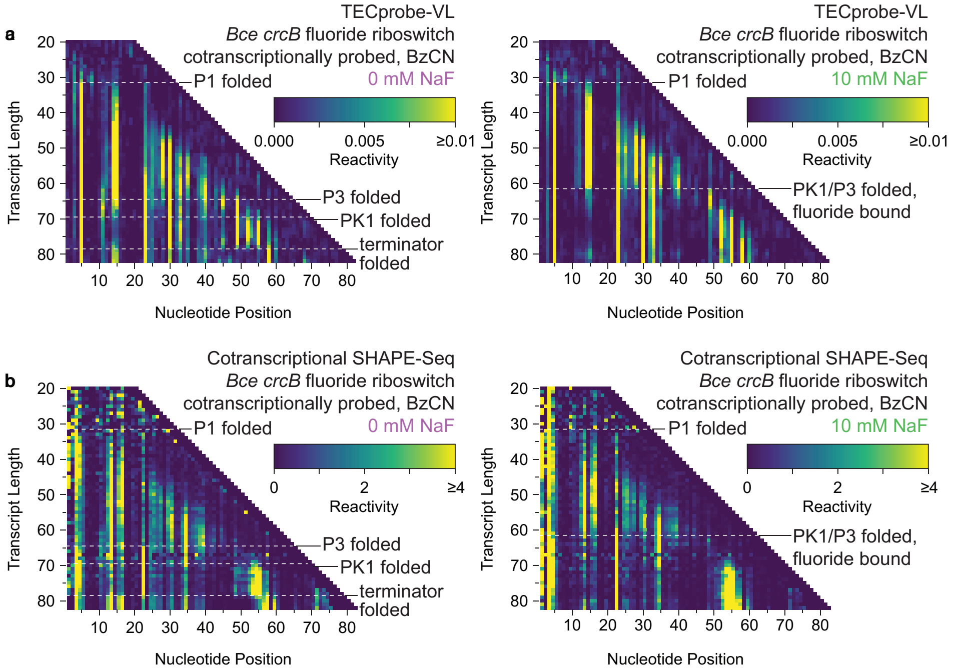

Supplementary Figure 14. Comparison of TECprobe-VL and Cotranscriptional SHAPE-Seq data: *B. cereus crcB* fluoride riboswitch reactivity matrices.
a, TECprobe-VL BzCN reactivity matrices for the *crcB* fluoride riboswitch with 0 mM and 10 mM NaF. Data are from Figure 4c, d and are presented here for comparison to cotranscriptional SHAPE-Seq data. **b**, Cotranscriptional SHAPE-Seq BzCN reactivity matrices for the *crcB* fluoride riboswitch with 0mM and 10 mM NaF. Cotranscriptional SHAPE-Seq data are from Strobel et al., 2017, *Distributed biotin-streptavidin transcription roadblocks for mapping cotranscriptional RNA folding*, *Nucleic Acids Res.* 2017 Jul; 45(12):e109 (Ref. 15). Source data are provided as a Source Data file. BzCN, benzoyl cyanide; PK1, pseudoknot.

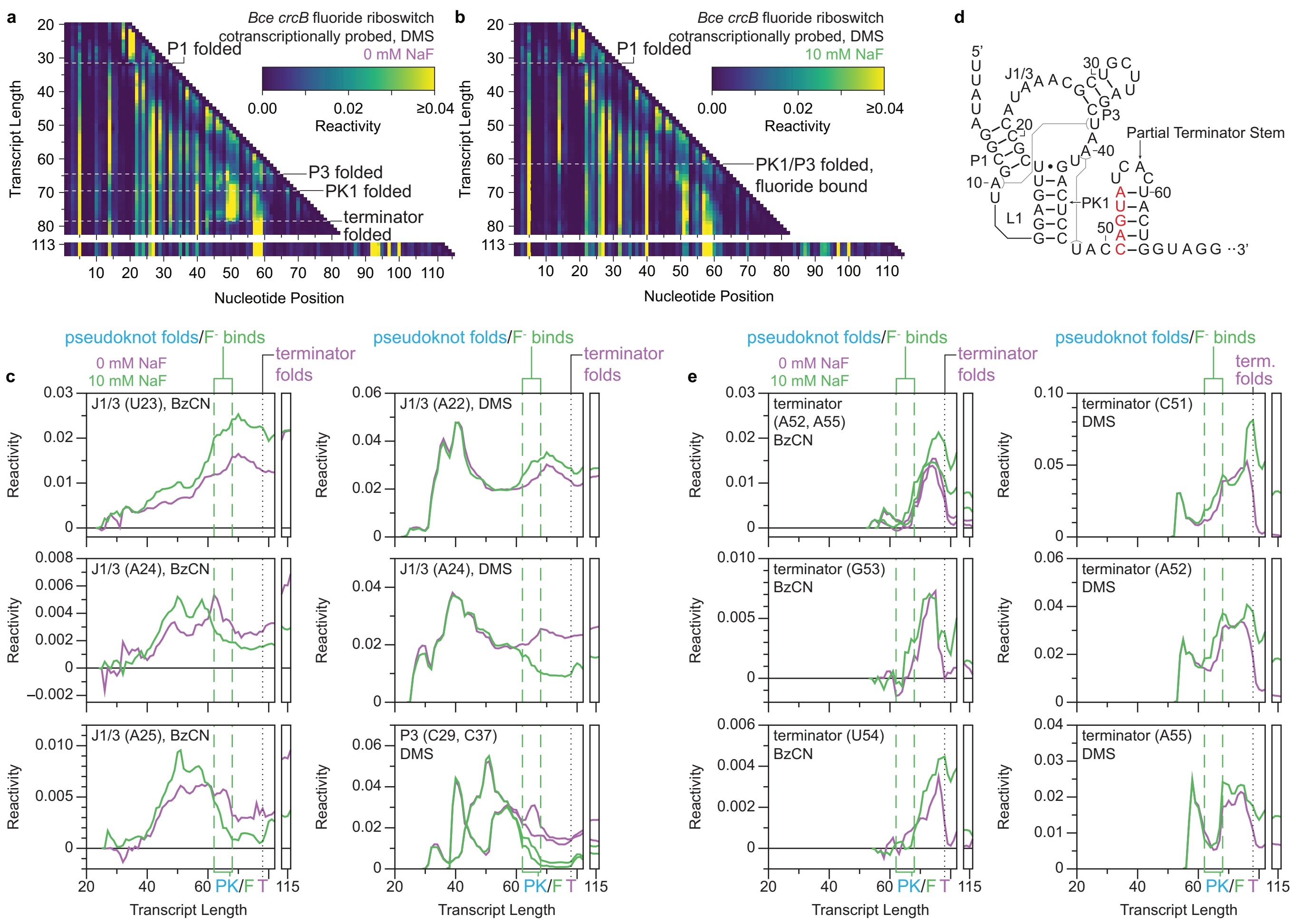

Supplementary Figure 15. Cotranscriptional DMS probing of the *B. cereus crcB* fluoride riboswitch and additional visualization of cotranscriptional folding transitions.
a, **b**, TECprobe-VL DMS reactivity matrices for the *Bce crcB* fluoride riboswitch with 0 mM and 10 mM NaF. Transcripts 83-112, which were not enriched, are excluded. Reactivity is shown as background-subtracted mutation rate. Data are from two independent replicates that were concatenated and analyzed together. **c**, Transcript length-dependent reactivity changes in J1/3 and P3. **d**, Secondary structure of the *crcB* fluoride riboswitch with a partially folded terminator stem. Red nucleotides exhibit fluoride-dependent reactivity changes that are consistent with delayed terminator hairpin nucleation. **e**, Transcript length-dependent reactivity changes in the terminator hairpin. Data from 0 mM and 10 mM NaF samples are purple and green, respectively. Vertical dotted and dashed lines mark when the indicated folding transitions occur. BzCN data are from Figure 4c, d. Source data are provided as a Source Data file. DMS, dimethyl sulfate; PK1, pseudoknot; BzCN, benzoyl cyanide.

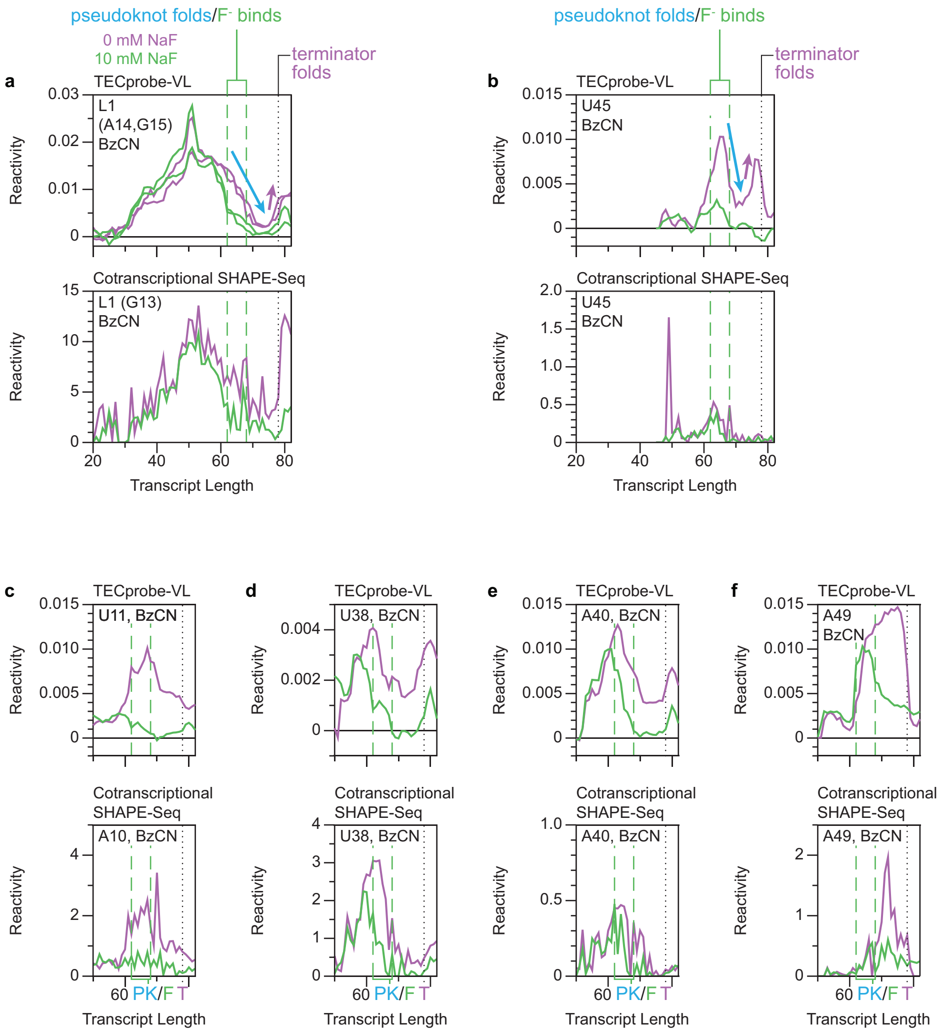

Supplementary Figure 16. Comparison of TECprobe-VL and Cotranscriptional SHAPE-Seq data: *B. cereus crcB* fluoride riboswitch pseudoknot formation/disruption and long-range base pair formation. Plots of TECprobe-VL (top) and Cotranscriptional SHAPE-Seq (bottom) transcript length-dependent BzCN reactivity changes showing pseudoknot formation/disruption (**a**, **b**) and long-range base pair formation (**c-f**). Data from 0 mM and 10 mM NaF samples are purple and green, respectively. TECprobe-VL data are from Figure 4c,d. Cotranscriptional SHAPE-Seq data are from Strobel et al., 2017, *Distributed biotin-streptavidin transcription roadblocks for mapping cotranscriptional RNA folding*, *Nucleic Acids Res.* 2017 Jul; 45(12):e109 (Ref. 15). Source data are provided as a Source Data file. BzCN, benzoyl cyanide; PK, pseudoknot folds; F, fluoride binds; T, terminator folds.

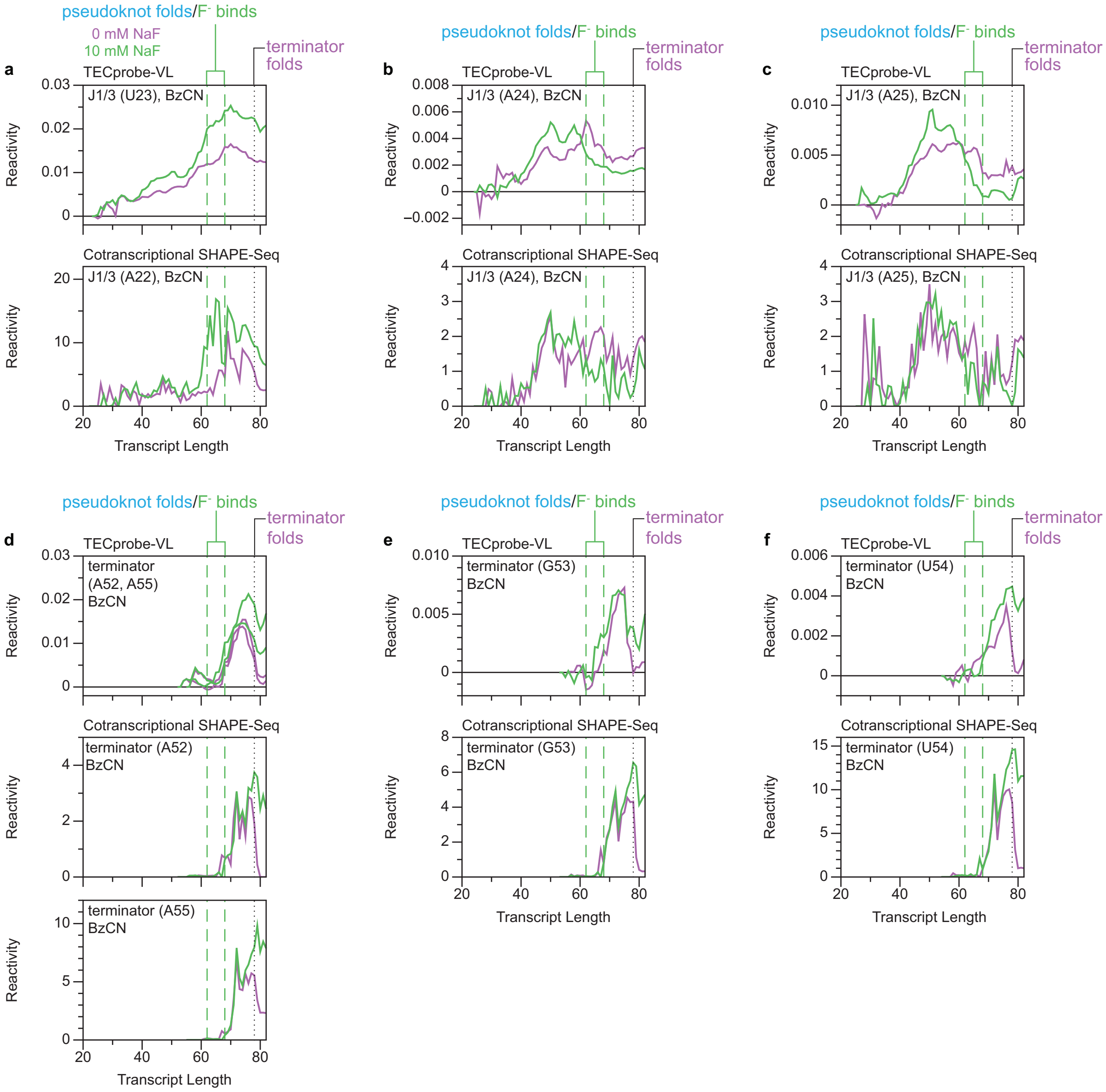

Supplementary Figure 17. Comparison of TECprobe-VL and Cotranscriptional SHAPE-Seq data: *B. cereus crcB* fluoride riboswitch J1/3 stabilization and terminator folding.

Plots of TECprobe-VL (top) and Cotranscriptional SHAPE-Seq (bottom) transcript length-dependent BzCN reactivity changes showing J1/3 stabilization (**a-c**) and terminator folding (**d-f**). Data from 0 mM and 10 mM NaF samples are purple and green, respectively. In panel d, reactivity trajectories that were plotted together for TECprobe-VL data are shown separately for Cotranscriptional SHAPE-Seq data to facilitate clear visualization of the data. TECprobe-VL data are from Figure 4c,d. Cotranscriptional SHAPE-Seq data are from Strobel et al., 2017, *Distributed biotin-streptavidin transcription roadblocks for mapping cotranscriptional RNA folding*, *Nucleic Acids Res.* 2017 Jul; 45(12):e109 (Ref. 15). Source data are provided as a Source Data file. BzCN, benzoyl cyanide.

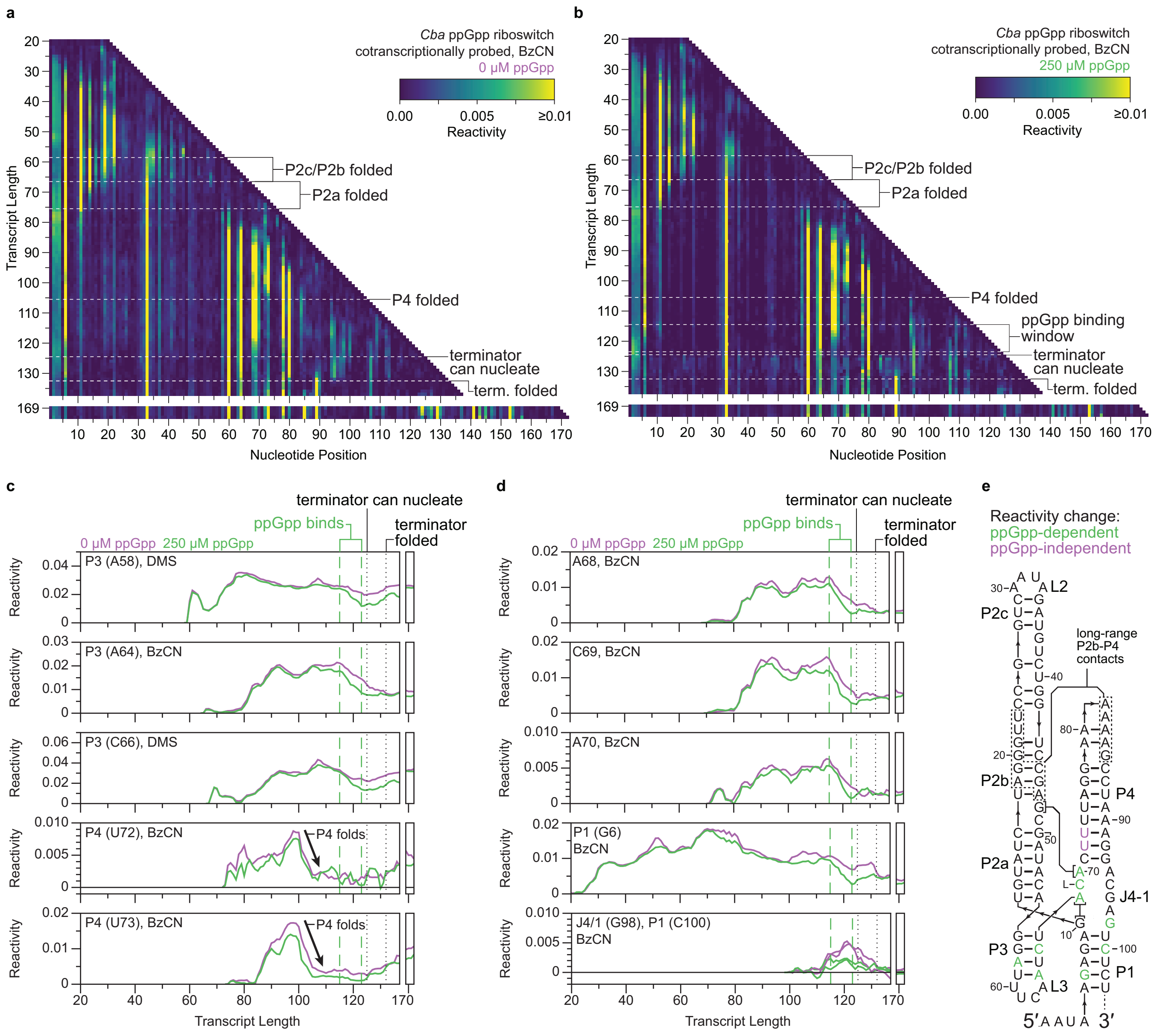

Supplementary Figure 18. Cotranscriptional BzCN probing and late folding transitions of the *C. bacterium* ppGpp riboswitch.

a, **b**, TECprobe-VL BzCN reactivity matrices for the *Cba* ppGpp riboswitch with 0 μM and 250 μM ppGpp. Transcripts 138-168, which were not enriched, are excluded. The annotation ‘terminator can nucleate’ indicates the transcript at which a coordinated weak increase in reactivity is observed at numerous positions in the ppGpp aptamer when ppGpp is present, which can be seen in panel b. Reactivity is shown as background-subtracted mutation rate. Data are from two independent replicates that were concatenated and analyzed together. **c**, Transcript length-dependent reactivity changes in P3 and P4. **d**, Transcript length-dependent reactivity changes in the ligand binding pocket, J1/4, and P1. Data from 0 μM and 250 μM ppGpp samples are purple and green, respectively**.** Vertical dotted and dashed lines mark when the indicated folding transitions occur. **e**, *Cba* ppGpp aptamer secondary structure showing the location of nucleotides that are highlighted in panels (**c**) and (**d**). Nucleotides colored green exhibit reduced reactivity upon ppGpp binding. Nucleotides colored purple decrease in reactivity upon P4 folding independent of ppGpp. Source data are provided as a Source Data file. BzCN, benzoyl cyanide.

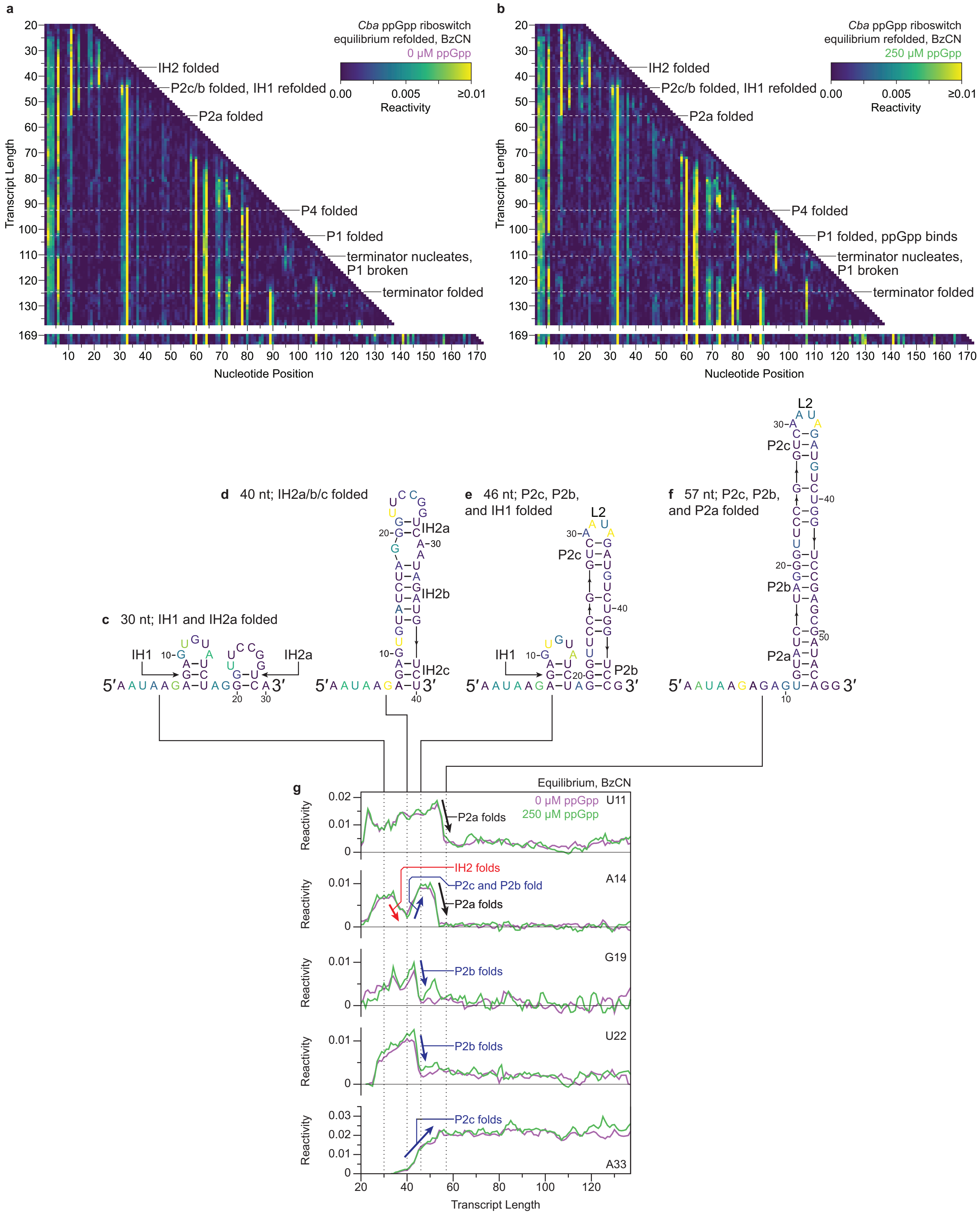

Supplementary Figure 19. Equilibrium BzCN probing of the *C. bacterium* ppGpp riboswitch.
a, **b**, TECprobe-VL BzCN reactivity matrices of *Cba* ppGpp riboswitch intermediate transcripts that were purified and refolded in the absence or presence of 250 μM ppGpp before chemical probing. Transcripts 138-168, which were not enriched, are excluded. Reactivity is shown as background-subtracted mutation rate. **c-f**,

Secondary structures of proposed early folding intermediates colored by reactivity from transcripts 30 (**c**), 40 (**d**), 46 (**e**), and 57 (**f**) in the absence of ppGpp. **g**, Plots showing transcript length-dependent reactivity changes that occur during P2 hairpin folding. Black dotted lines indicate the transcript lengths used to identify the intermediate structures shown in **c-f**. Arrows indicate reactivity changes associated with P2a (black), P2b/P2c (blue), and IH2 (red) folding. Data from 0 μM and 250 μM ppGpp samples are purple and green, respectively. Source data are provided as a Source Data file. BzCN, benzoyl cyanide.

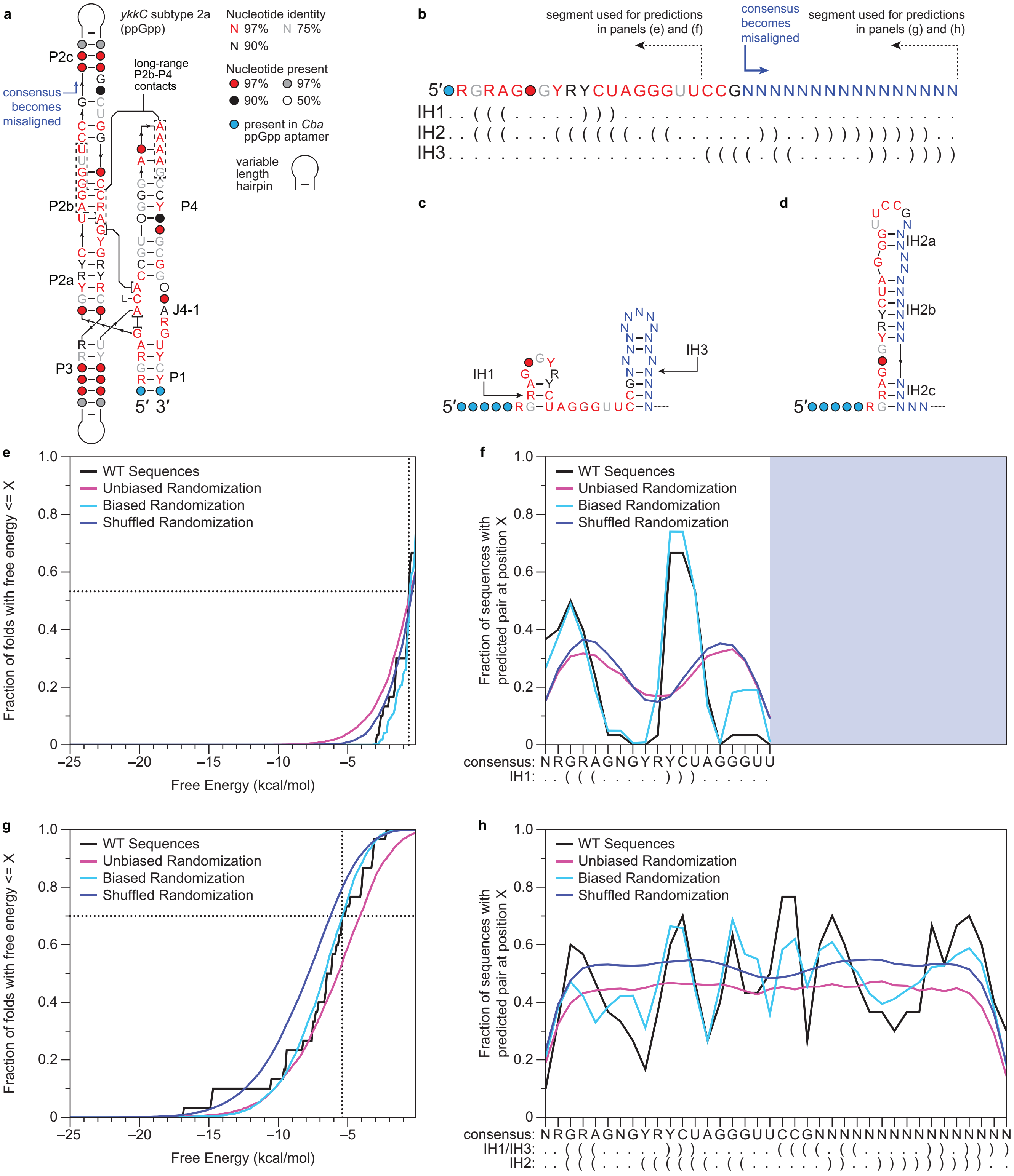

Supplementary Figure 20. Analysis of ppGpp riboswitch sequences for capacity to form intermediate hairpins.

a, ppGpp aptamer consensus structure adapted from Sherlock et al. (2018)[23] and Knappenberger et al.(2018)[47]. The location at which ppGpp aptamer sequences are no longer aligned due to a variable length hairpin is annotated. **b**, Diagram of the ppGpp aptamer segments used to perform the minimum free energy structure predictions in panels **e-h**. **c, d**, IH1/IH3 (**c**) and IH2 (**d**) secondary structures drawn using the ppGpp

aptamer consensus sequence. **e, g**, Cumulative distribution plots of the minimum free energy (ΔG) predicted for ZTP riboswitch sequences that correspond to the *Cba* ppGpp riboswitch intermediate hairpin structures. Structure prediction was performed using the RNAstructure[49] Fold command. The analysis of several sequence pools is presented: 30 ppGpp riboswitches from fully sequenced bacterial genomes (black); 3000 sequences generated with an equal probability for observing each nucleotide at each position (magenta); 3000 sequences randomized by the natural nucleotide frequency at each position (blue); 500 sequences per riboswitch (15000 total) randomized by shuffling nucleotide position (purple). The intersection of the dotted lines marks the location of the *Cba* ppGpp riboswitch within the plot. In some wild-type sequences the variable length hairpin was sufficiently short P2c was able to fold and therefore contribute to the ΔG of the minimum free energy structure. In these cases, sequence that preceded P2c always formed a non-native structure in addition to the native helix. **f, h**, Plots showing the fraction of structure predictions from panel e (**f**) and panel g (**h**) with a predicted base pair at each position of the sequence. Source data are provided as a Source Data file.

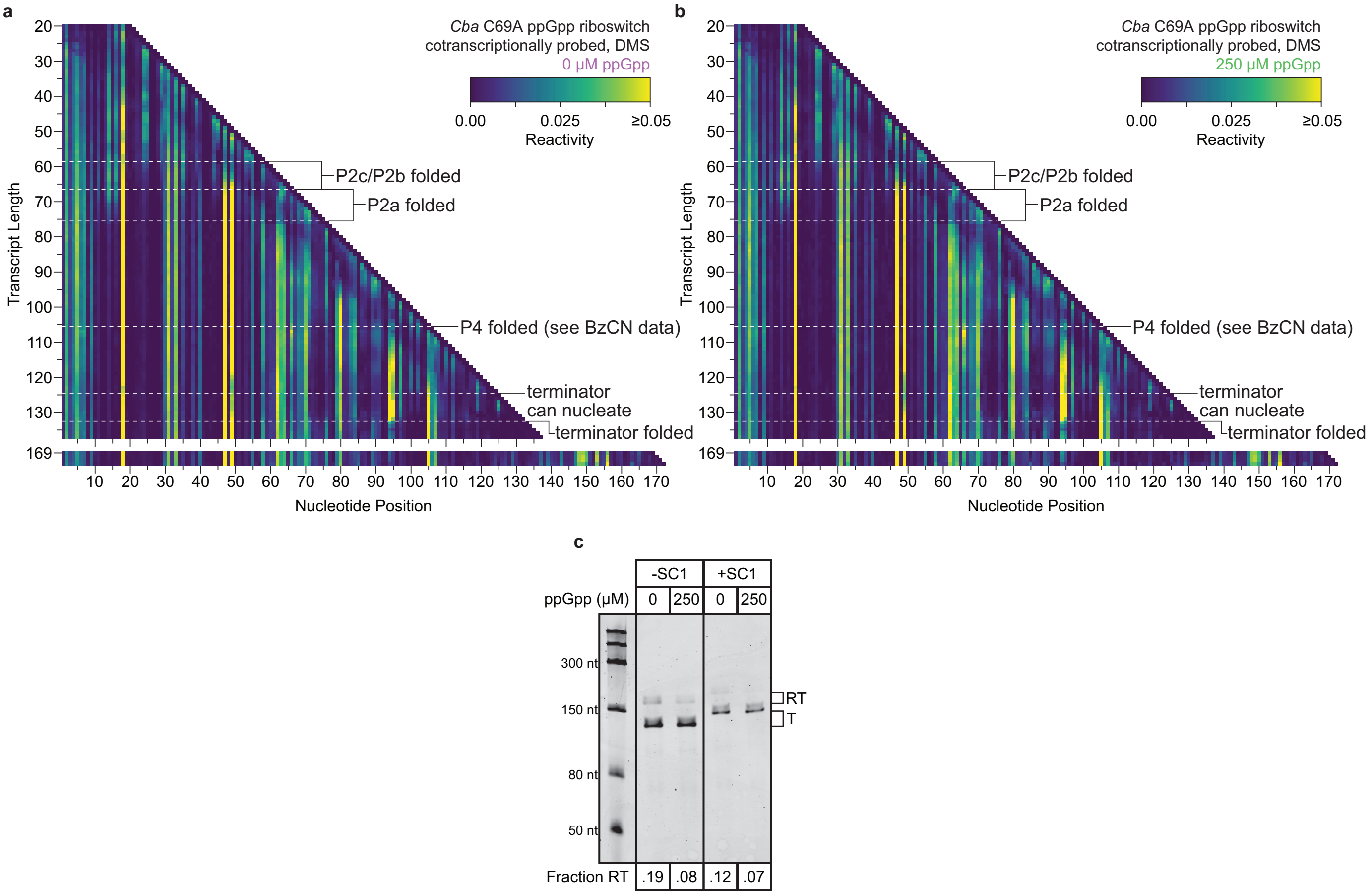

Supplementary Figure 21. Cotranscriptional DMS probing of the *C. bacterium* ppGpp riboswitch C69A variant.

a, **b**, TECprobe-VL DMS reactivity matrices for the *Cba* ppGpp riboswitch C69A variant with 0 µM and 250 µM ppGpp. Transcripts 138-168, which were not enriched, are excluded. Data are from two independent replicates that were concatenated and analyzed together. Reactivity is shown as background-subtracted mutation rate. **c**, Single-round transcription termination assays for the *Cba* ppGpp riboswitch C69A variant with or without SC1. Fraction readthrough is the average of two independent replicates. This experiment was run on the same gel as Figure 5c and therefore has the same ladder. An uncropped gel image is provided in Supplementary Fig. 24. Source data are provided as a Source Data file. DMS, dimethyl sulfate; BzCN, benzoyl cyanide; SC1, structure cassette 1; RT, readthrough; T, terminated.

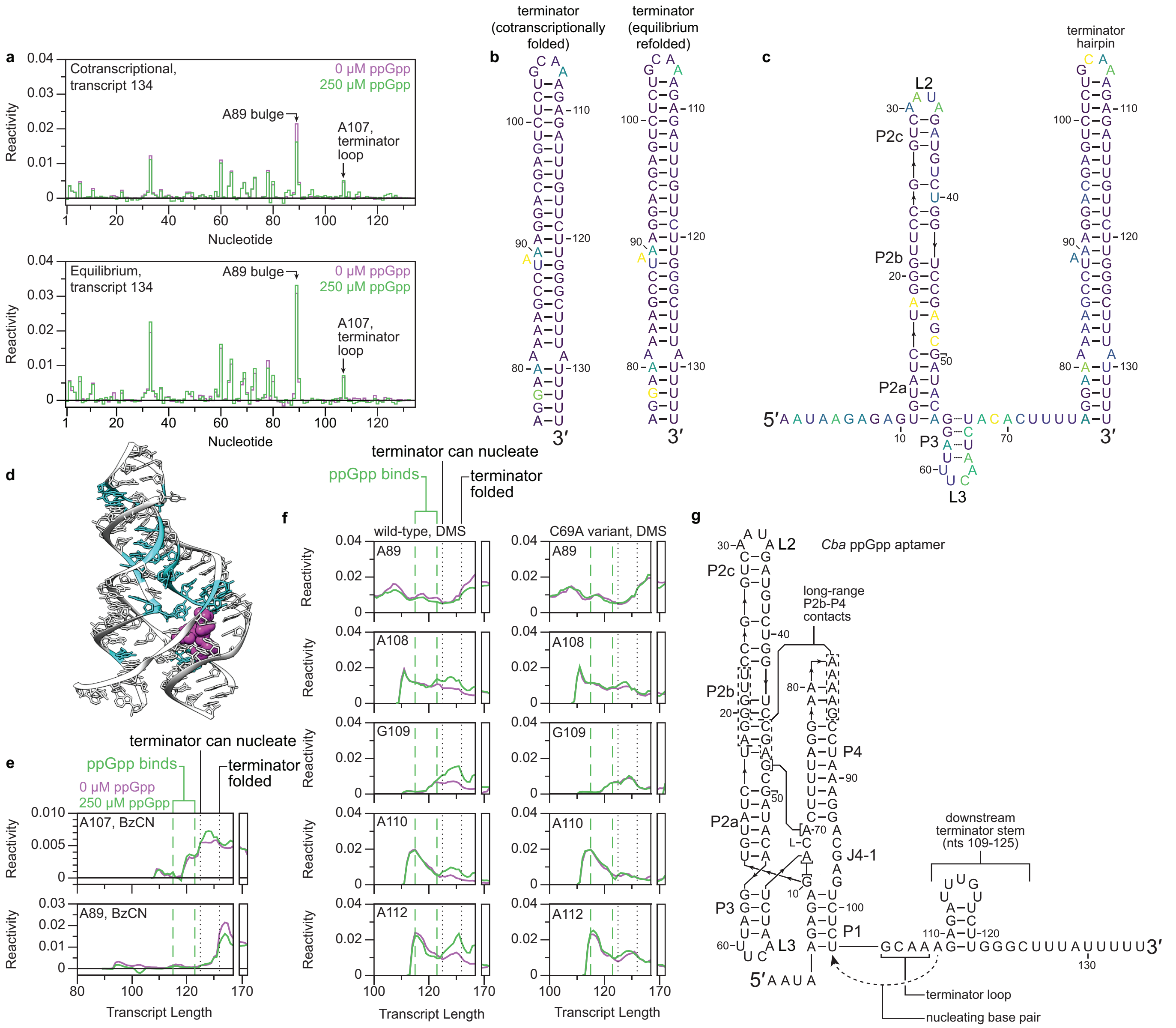

Supplementary Figure 22. Visualization of *Cba* ppGpp terminator hairpin folding.
a, BzCN reactivity of *Cba* ppGpp riboswitch transcripts from the primary transcription termination site (nt 134) in cotranscriptional (top) and equilibrium (bottom) probing conditions. **b**,Terminator hairpin secondary structures colored by the cotranscriptional (left) and equilibrium (right) BzCN reactivity of transcript 134 from the 0 mM ppGpp data sets. **c**, Secondary structure of the terminated *Cba* ppGpp riboswitch colored by the 0µM ppGpp DMS reactivity of transcript 134. **d**, Structure of the *Thermoanaerobacter mathranii* PRPP riboswitch G96A (ppGpp-binding) variant (PDB 6CK4)[47] highlighted to indicate nucleotides that undergo a weak increase in BzCN reactivity at transcript 125 in the presence of ppGpp. **e**, Transcript length-dependent BzCN reactivity changes that occur during cotranscriptional terminator folding. **f**, Transcript length-dependent DMS reactivity changes in terminator nucleotides. **g**, *Cba* ppGpp riboswitch secondary structure showing a predicted alternative structure within the downstream terminator stem. Data from 0 µM and 250 µM ppGpp samples are purple and green, respectively. Vertical dotted and dashed lines mark when the indicated folding transitions occur. Data are from Figure 5d, e and Supplementary Figures 18a, 18b, 19a, 19b, 21a, and 21b. Source data are provided as a Source Data file. BzCN, benzoyl cyanide; DMS, dimethyl sulfate.

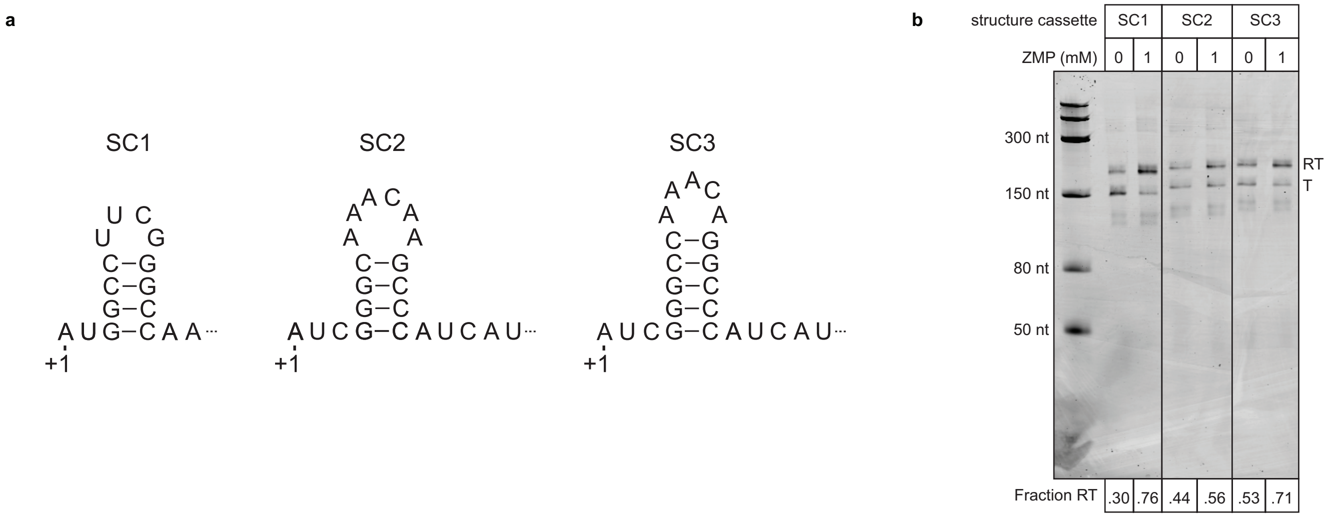

Supplementary Figure 23. Comparison of 5' structure cassettes.
(a) Secondary structures of three structure cassettes that were assessed. **(b)** Single-round transcription termination assays for the *pfl* ZTP riboswitch with the structures cassettes shown in (a) in the presence and absence of ZMP. Source data are provided as a Source Data file.

Supplementary Figure 24. Uncropped source gels. Uncropped source gels are provided for all gels shown in main and supplemental figures. Boxes indicate the approximate included area in the figures.

Uncropped source gels for quantification in Figure 3b
Replicate 2 is shown as a reprentative gel in Figure 3b

Figure 3b Replicate 1
(only quantification is shown)

Figure 3b Replicate 2

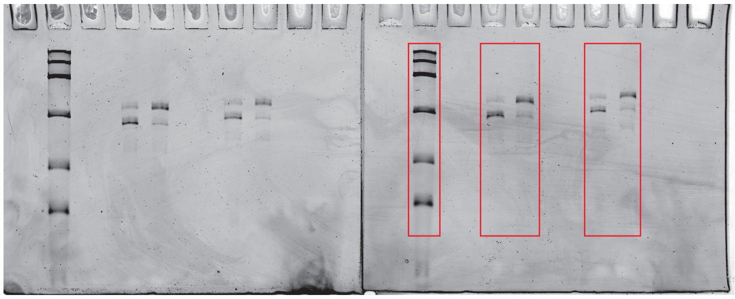

Uncropped source gels for quantification in Figure 4b
Replicate 2 is shown as a reprentative gel in Figure 4b

Figure 4b Replicate 1
(only quantification is shown)

Figure 4b Replicate 2

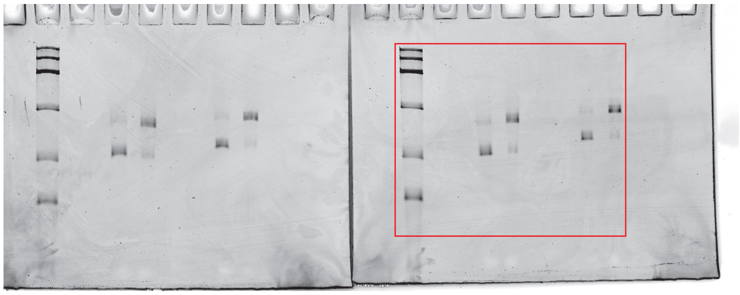

Uncropped source gels for quantification in Figure 5c and Supplementary Figure 21c
Replicate 3 is shown as a reprentative gel in Figure 5c and Supplementary Figure 21c
The same ladder is shown in both figures; Omitted lanes contained 1mM pAp

Figure 5c Rep 1 and
Supplementary Figure 21c Rep 1
(only quantification is shown)

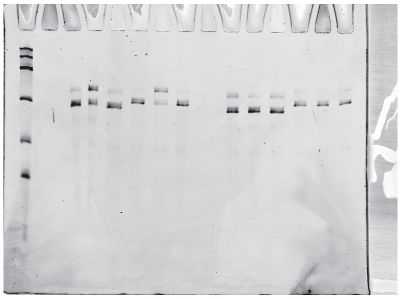

Figure 5c Rep 2 (only quantification is shown)
and C69U variant replicate 1 (not shown)

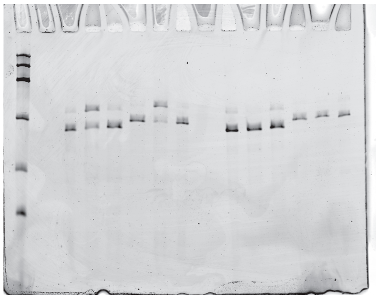

Figure 5c Rep3 and
Supplementary Figure 21c Rep 2

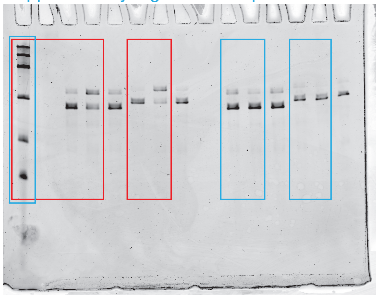

Figure 5c Rep 4 (only quantification is shown)
and C69U variant replicate 2 (not shown)

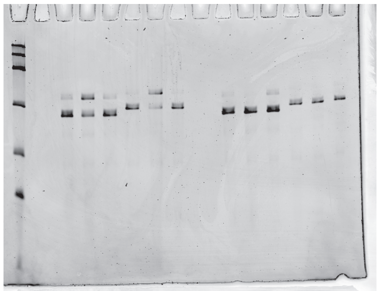

(Supplementary Figure 24 continues on the next page)

Supplementary Figure 24 (continued)

Uncropped source gel for Supp. Fig. 2

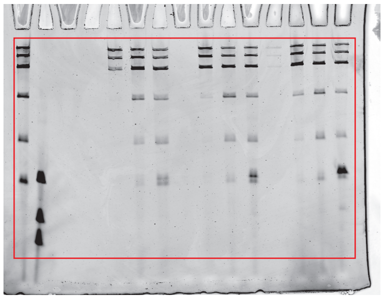

Uncropped source gel for Supp. Fig. 3a

Uncropped source gel for Supp. Fig. 3c, left gel

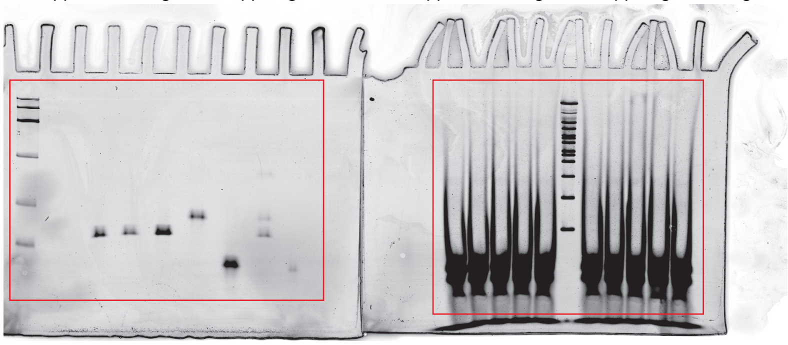

Unrelated gel that was scanned at the same time as the Supp. Fig 3c. source gel

Uncropped source gel for Supp. Fig. 3c, right gel

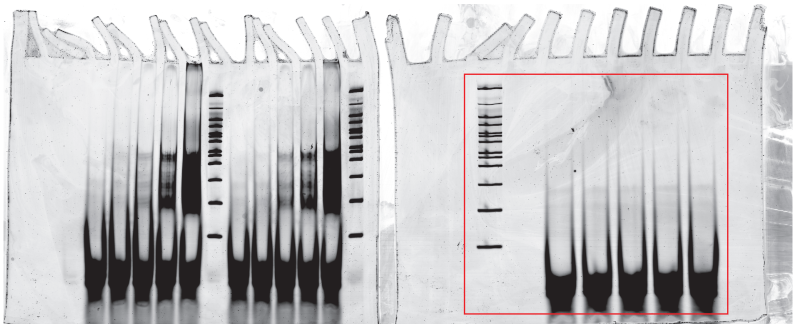

Uncropped source gel for Supp. Fig. 4a

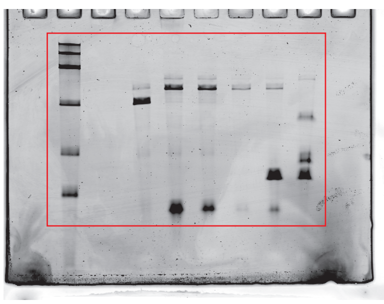

Uncropped source gel for Supp. Fig. 4b

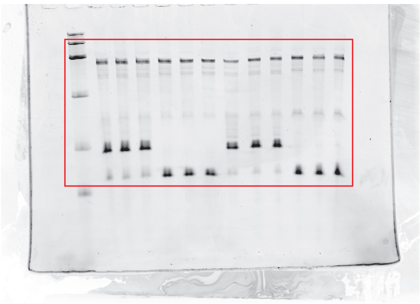

(Supplementary Figure 24 continues on the next page)

Supplementary Figure 24 (continued)

Uncropped source gel for Supp. Fig. 4c

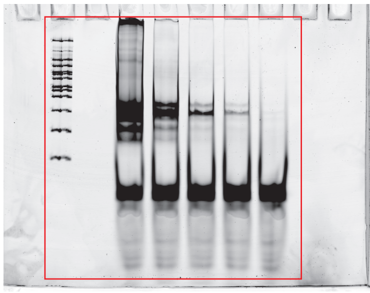

Same samples as Supp. Fig. 4c but analyzed by denaturing PAGE (not shown)

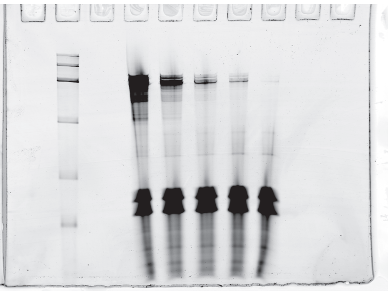

Uncropped source gel for Supp. Fig. 4d

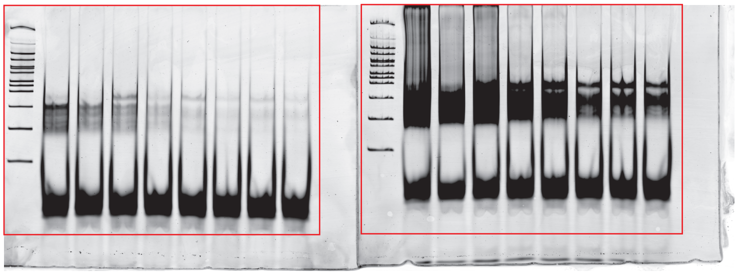

Unrelated gel that was scanned at the same time as the Supp. Fig. 23 source gel

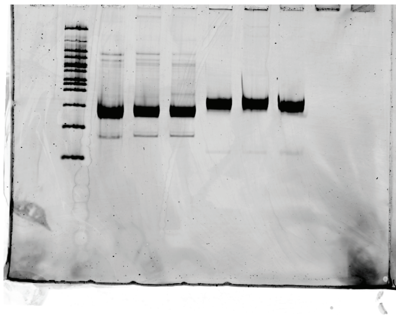

Uncropped source gel for Supp. Fig 23
The lanes immediately to the right of the included lanes contain +ZMP samples that were processed by phenol/chloroform extraction (instead of TRIzol) and without DNase I treatment. The 5 lanes to the right contain DNA templates.

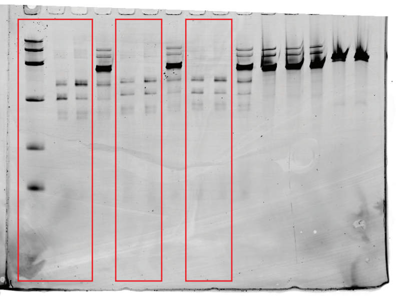

Supplementary Table 1. Oligonucleotides used in this study. The table below shows oligonucleotides used in this study. The modification codes presented are compatible with Integrated DNA Technology ordering.

```
/iBioTEG/: internal biotin-triethylene glycol
/iEth-dA/: internal etheno-dA
 /5bioSG/: “standard” 5’ biotin
  /5Phos/: 5’ phosphate
  /3AmMO/: 3’ amino modifier
```

ID	Name	Sequence	Purification
TECD001	dRP1iBio.R	AATGATACGGCGACCACCGAGATCTACAC/iBioTEG/GTTCAGAGTTCTACAGTCCGAC GATC	HPLC
TECD002	dRP1iEthDA.R	AATGATACGGCGACCACCGAGATCTACAC/iEth-dA/GTTCAGAGTTCTACAGTCCGACGATC	HPLC
TECD006	PRA1_NoMod.F	TTATCAAAAAGAGTATTGACTCTTTTACCTCTGGCGGTGATAATGGTTGCAT	HPLC
TECD017	dRP1_NoMod.R	AATGATACGGCGACCACCGAGATCTACACGTTCAGAGTTCTACAGTCCGACGATC	HPLC
STD000	pRA1_shrt.F	GAGGCTTTACACTTTATGCTTCCGGCTG	none
STD002	HP4_5bio.R	/5Biosg/AATGTCTTCCAGCACACATCGCCTGACGAATCA	none
STD003	dRP1_5bio.R	/5Biosg/AATGATACGGCGACCACCGAGATCTACACGTTCAGAGTTCTACAGTCCGACG ATC	HPLC
TECP001	9N_VRA3	/5Phos/rNrNrNrNrNrNNNNGATCGTCGGACTGTAGAACTCTGAAC/3AmMO/	HPLC
TECP002	SC1Brdg_MINUS	CCTTGGCACCCGAGAATTCCAYYYRRATGGCCTTCGGGCCAA	HPLC
TECP003	SC1Brdg_PLUS	CCTTGGCACCCGAGAATTCCARRRYYATGGCCTTCGGGCCAA	HPLC
RPIX_SC1	RPIX_SC1_Bridge	CCTTGGCACCCGAGAATTCCAATGGCCTTCGGGCCAA	none
RPIX	RPIX	CAAGCAGAAGACGGCATACGAGATCGTGATGTGACTGGAGTTCCTTGGCACCCGAG AATTCCA	none
RPI	RPI	CAAGCAGAAGACGGCATACGAGAT[Index]GTGACTGGAGTTCCTTGGCACCCGAGAA TTCCA	PAGE

Supplementary Table 2. DNA templates prepared for this study. The table below describes the DNA templates prepared for this study, including the primers and templates used, DNA modifications, whether translesion synthesis was performed, how the DNA template was purified, and the figures in which each DNA templates was used

ID	Fwd Primer	Rev Primer	Template	Modifications	Translesion Synthesis	Clean up	Used in Fig(s)
1	TECD006	STD002	pCES007	5' biotin	N/A	Gel extracted	3
2	TECD006	STD002	pCES008	5' biotin	N/A	Gel extracted	3, S23
3	TECD006	STD002	Template 2	Internal biotin-11 nucleotides, 5' biotin	N/A	SPRI beads	1, 2, 3, S1, S5, S6, S7, S8, S9, S10, S11, S12
4	TECD006	STD002	pCES009	5' biotin	N/A	Gel extracted	4
5	TECD006	STD002	pCES010	5' biotin	N/A	Gel extracted	4
6	TECD006	STD002	Template 5	Internal biotin-11 nucleotides, 5' biotin	N/A	SPRI beads	2, 4, S1, S4, S5, S7, S14, S15, S16 S17
7	STD000	STD002	pCES011	5' biotin	N/A	Gel extracted	5
8	STD000	STD002	pCES012	5' biotin	N/A	Gel extracted	5, S3
9	STD000	STD002	Template 8	Internal biotin-11 nucleotides, 5' biotin	N/A	SPRI beads	2, 5, 6, 7, S1, S4, S5, S6, S18, S19, S21, S22
10	STD000	STD002	pCES013	5' biotin	N/A	Gel extracted	S21
11	STD000	STD002	pCES014	5' biotin	N/A	Gel extracted	S21
12	STD000	STD002	Template 11	Internal biotin-11 nucleotides, 5' biotin	N/A	SPRI beads	2, 7, S21, S22
13	TECD006	TECD002	Gel-purified linear template from pCES015	Internal etheno-dA	Yes	SPRI beads	S13
14	TECD006	TECD001	Gel-purified linear template from pCES015	Internal Bio-TEG	Yes	SPRI beads	S4
15	TECD006	STD002	pCES016	5' biotin	N/A	Gel extracted	S23
16	TECD006	STD002	pCES017	5' biotin	N/A	Gel extracted	S23

Supplementary Table 3. DNA template sequences. The table below contains the DNA sequences used in this study.

Name	Sequence
PRA1_CbePfl_HP4	ttatcaaaaagagtattgactcttttacctctggcggtgataatggttgcatattagatattagtcatatgactgacggaagtggagttaccacatgaagtatgactaggcatattatcttatatgccacaaaaagccgaccgtctgggcaaaaaaagcctggattgcgtcggcttttttatatggaaacctgattcgtcaggcgatgtgtgctggaagacatt
PRA1_SC1_CbePfl_HP4	ttatcaaaaagagtattgactcttttacctctggcggtgataatggttgcatggccttcgggccaaattagatattagtcatatgactgacggaagtggagttaccacatgaagtatgactaggcatattatcttatatgccacaaaaagccgaccgtctgggcaaaaaaagcctggattgcgtcggcttttttatatggaaacctgattcgtcaggcgatgtgtgctggaagacatt
PRA1_Bce_crcB_HP4	ttatcaaaaagagtattgactcttttacctctggcggtgataatggttgcatttataggcgatggagttcgccataaacgctgcttagctaatgactcctaccagtatcactactggtaggagtctatttttttgagcaagctacctgattcgtcaggcgatgtgtgctggaagacatt
PRA1_SC1_Bce_crcB_HP4	ttatcaaaaagagtattgactcttttacctctggcggtgataatggttgcatggccttcgggccaattataggcgatggagttcgccataaacgctgcttagctaatgactcctaccagtatcactactggtaggagtctatttttttgagcaagctacctgattcgtcaggcgatgtgtgctggaagacatt
PRA1_Cba_HP4	gaggctttacactttatgcttccggctgaattctaaagatctttatcaaaaagagtattgactcttttacctctggcggtgataatggttgcataataagagagtgtatctagggttccggtcaatagatgtctggtccgagcgatacaggatttcaatctacacttttaggaaaaaagcctaaaggacgagtctctgcaaagagatttgttcttgggctttattttttatctttatttaacctgattcgtcaggcgatgtgtgctggaagacatt
PRA1_SC1_Cba_HP4	gaggctttacactttatgcttccggctgaattctaaagatctttatcaaaaagagtattgactcttttacctctggcggtgataatggttgcatggccttcgggccaaaataagagagtgtatctagggttccggtcaatagatgtctggtccgagcgatacaggatttcaatctacacttttaggaaaaaagcctaaaggacgagtctctgcaaagagatttgttcttgggctttattttttatctttatttaacctgattcgtcaggcgatgtgtgctggaagacatt
PRA1_Cba_C69A_HP4	gaggctttacactttatgcttccggctgaattctaaagatctttatcaaaaagagtattgactcttttacctctggcggtgataatggttgcataataagagagtgtatctagggttccggtcaatagatgtctggtccgagcgatacaggatttcaatctaaacttttaggaaaaaagcctaaaggacgagtctctgcaaagagatttgttcttgggctttattttttatctttatttaacctgattcgtcaggcgatgtgtgctggaagacatt
PRA1_SC1_C69A_HP4	gaggctttacactttatgcttccggctgaattctaaagatctttatcaaaaagagtattgactcttttacctctggcggtgataatggttgcatggccttcgggccaaaataagagagtgtatctagggttccggtcaatagatgtctggtccgagcgatacaggatttcaatctaaacttttaggaaaaaagcctaaaggacgagtctctgcaaagagatttgttcttgggctttattttttatctttatttaacctgattcgtcaggcgatgtgtgctggaagacatt
PRA1_SC1_CbePfl_NoPU_BcSeq_dRP1	ttatcaaaaagagtattgactcttttacctctggcggtgataatggttgcatggccttcgggccaaattagatattagtcatatgactgacggaagtggagttaccacatgaagtatgactaggcatattatcttatatgccacaaaaagccgaccgtctgggcaaaaaaagcctggattgcgtcggcaaaccaacctagtccttcgggactagcctttgatggtcgttcgcgatcgtcggactgtagaactctgaacgtgtagatctcggtggtcgccgtatcatt
PRA1_SC2_CbePfl_HP4	ttatcaaaaagagtattgactcttttacctctggcggtgataatggttgcatcgggcaaacaagcccatcatattagatattagtcatatgactgacggaagtggagttaccacatgaagtatgactaggcatattatcttatatgccacaaaaagccgaccgtctgggcaaaaaaagcctggattgcgtcggcttttttatatggaaaacctgattcgtcaggcgatgtgtgctggaagacatt
PRA1_SC3_CbePfl_HP4	ttatcaaaaagagtattgactcttttacctctggcggtgataatggttgcatcgggccaaacaggcccatcatattagatattagtcatatgactgacggaagtggagttaccacatgaagtatgactaggcatattatcttatatgccacaaaaagccgaccgtctgggcaaaaaaagcctggattgcgtcggcttttttatatggaaaacctgattcgtcaggcgatgtgtgctggaagacatt

Supplementary Table 4. Sequencing Read Archive (SRA) deposition table. All primary sequencing data generated in this work are freely available from the Sequencing Read Archive (http://www.ncbi.nlm.nih.gov/sra), accessible via the BioProject accession number PRJNA929456 or using the individual accession numbers below.

SRA Acession	Sample Name	Experiment
SAMN32973014	ZTP_BzCN_0mM_rep1	wt ZTP riboswitch multilength cotranscriptional BzCN probing with 0mM ZMP, replicate 1
SAMN32973015	ZTP_BzCN_0mM_rep2	wt ZTP riboswitch multilength cotranscriptional BzCN probing with 0mM ZMP, replicate 2
SAMN32973016	ZTP_BzCN_1mM_rep1	wt ZTP riboswitch multilength cotranscriptional BzCN probing with 1mM ZMP, replicate 1
SAMN32973017	ZTP_BzCN_1mM_rep2	wt ZTP riboswitch multilength cotranscriptional BzCN probing with 1mM ZMP, replicate 2
SAMN32973018	ZTP_DMS_0mM_rep1	wt ZTP riboswitch multilength cotranscriptional DMS probing with 0mM ZMP, replicate 1
SAMN32973019	ZTP_DMS_0mM_rep2	wt ZTP riboswitch multilength cotranscriptional DMS probing with 0mM ZMP, replicate 2
SAMN32973020	ZTP_DMS_1mM_rep1	wt ZTP riboswitch multilength cotranscriptional DMS probing with 1mM ZMP, replicate 1
SAMN32973021	ZTP_DMS_1mM_rep2	wt ZTP riboswitch multilength cotranscriptional DMS probing with 1mM ZMP, replicate 2
SAMN32973022	ZTP_SL_BzCN_0mM_lo_rep1	wt ZTP riboswitch single length cotranscriptional BzCN probing with 0mM ZMP and 100 uM NTPs, replicate 1
SAMN32973023	ZTP_SL_BzCN_0mM_lo_rep2	wt ZTP riboswitch single length cotranscriptional BzCN probing with 0mM ZMP and 100 uM NTPs, replicate 2
SAMN32973024	ZTP_SL_BzCN_0mM_hi_rep1	wt ZTP riboswitch single length cotranscriptional BzCN probing with 0mM ZMP and 500 uM NTPs, replicate 1
SAMN32973025	ZTP_SL_BzCN_0mM_hi_rep2	wt ZTP riboswitch single length cotranscriptional BzCN probing with 0mM ZMP and 500 uM NTPs, replicate 2
SAMN32973026	ZTP_SL_BzCN_1mM_lo_rep1	wt ZTP riboswitch single length cotranscriptional BzCN probing with 1mM ZMP and 100 uM NTPs, replicate 1
SAMN32973027	ZTP_SL_BzCN_1mM_lo_rep2	wt ZTP riboswitch single length cotranscriptional BzCN probing with 1mM ZMP and 100 uM NTPs, replicate 2
SAMN32973028	ZTP_SL_BzCN_1mM_hi_rep1	wt ZTP riboswitch single length cotranscriptional BzCN probing with 1mM ZMP and 500 uM NTPs, replicate 1
SAMN32973029	ZTP_SL_BzCN_1mM_hi_rep2	wt ZTP riboswitch single length cotranscriptional BzCN probing with 1mM ZMP and 500 uM NTPs, replicate 2
SAMN32973030	F_BzCN_00mM_rep1	wt fluoride riboswitch multilength cotranscriptional BzCN probing with 0 mM NaF, replicate 1
SAMN32973031	F_BzCN_00mM_rep2	wt fluoride riboswitch multilength cotranscriptional BzCN probing with 0 mM NaF, replicate 2
SAMN32973032	F_BzCN_10mM_rep1	wt fluoride riboswitch multilength cotranscriptional BzCN probing with 10 mM NaF, replicate 1
SAMN32973033	F_BzCN_10mM_rep2	wt fluoride riboswitch multilength cotranscriptional BzCN probing with 10 mM NaF, replicate 2
SAMN32973034	F_DMS_00mM_rep1	wt fluoride riboswitch multilength cotranscriptional DMS probing with 0 mM NaF, replicate 1
SAMN32973035	F_DMS_00mM_rep2	wt fluoride riboswitch multilength cotranscriptional DMS probing with 0 mM NaF, replicate 2
SAMN32973036	F_DMS_10mM_rep1	wt fluoride riboswitch multilength cotranscriptional DMS probing with 10 mM NaF, replicate 1
SAMN32973037	F_DMS_10mM_rep2	wt fluoride riboswitch multilength cotranscriptional DMS probing with 10 mM NaF, replicate 2
SAMN32973038	G4P_BzCN_000uM_rep1	wt ppGpp riboswitch multilength cotranscriptional BzCN probing with 0 uM ppGpp, replicate 1
SAMN32973039	G4P_BzCN_000uM_rep2	wt ppGpp riboswitch multilength cotranscriptional BzCN probing with 0 uM ppGpp, replicate 2
SAMN32973040	G4P_BzCN_250uM_rep1	wt ppGpp riboswitch multilength cotranscriptional BzCN probing with 250 uM ppGpp, replicate 1
SAMN32973041	G4P_BzCN_250uM_rep2	wt ppGpp riboswitch multilength cotranscriptional BzCN probing with 250 uM ppGpp, replicate 2

Supplementary Table 4 (continued)

SRA Acession	Sample Name	Experiment
SAMN32973042	G4P_DMS_000uM_rep1	wt ppGpp riboswitch multilength cotranscriptional DMS probing with 0 uM ppGpp, replicate 1
SAMN32973043	G4P_DMS_000uM_rep2	wt ppGpp riboswitch multilength cotranscriptional DMS probing with 0 uM ppGpp, replicate 2
SAMN32973044	G4P_DMS_250uM_rep1	wt ppGpp riboswitch multilength cotranscriptional DMS probing with 250 uM ppGpp, replicate 1
SAMN32973045	G4P_DMS_250uM_rep2	wt ppGpp riboswitch multilength cotranscriptional DMS probing with 250 uM ppGpp, replicate 2
SAMN32973046	G4P_EQ_BzCN_000uM_rep1	wt ppGpp riboswitch multilength equilibrium BzCN probing with 0 uM ppGpp, replicate 1
SAMN32973047	G4P_EQ_BzCN_250uM_rep1	wt ppGpp riboswitch multilength equilibrium BzCN probing with 250 uM ppGpp, replicate 1
SAMN32973048	G4PC69A_DMS_000uM_rep1	ppGpp riboswitch C69A variant multilength cotranscriptional DMS probing with 0 uM ppGpp, replicate 1
SAMN32973049	G4PC69A_DMS_000uM_rep2	ppGpp riboswitch C69A variant multilength cotranscriptional DMS probing with 0 uM ppGpp, replicate 2
SAMN32973050	G4PC69A_DMS_250uM_rep1	ppGpp riboswitch C69A variant multilength cotranscriptional DMS probing with 250 uM ppGpp, replicate 1
SAMN32973051	G4PC69A_DMS_250uM_rep2	ppGpp riboswitch C69A variant multilength cotranscriptional DMS probing with 250 uM ppGpp, replicate 2

Supplementary Table 5. RMDB data deposition table.
Reactivity data generated in this work are freely available from the RNA Mapping Database (RMDB)[81] (http://rmdb.stanford.edu), accessible using the RMDB ID numbers indicated in the table below.

RMDB Accession	RNA	Experiment	Folding	Probe	Ligand	NTP Conc.	Concatenated	Smoothing
CBAG4P_BZCN_0001	wt ppGpp riboswitch	TECprobe-VL	Cotranscriptional	BzCN	none	100uM	Reps 1 and 2	Yes
CBAG4P_BZCN_0002	wt ppGpp riboswitch	TECprobe-VL	Cotranscriptional	BzCN	250uM ppGpp	100uM	Reps 1 and 2	Yes
CBAG4P_BZCN_0003	wt ppGpp riboswitch	TECprobe-VL	Equilibrium	BzCN	none	100uM	No	Yes
CBAG4P_BZCN_0004	wt ppGpp riboswitch	TECprobe-VL	Equilibrium	BzCN	250uM ppGpp	100uM	No	Yes
CBAG4P_DMS_0001	wt ppGpp riboswitch	TECprobe-VL	Cotranscriptional	DMS	none	100uM	Reps 1 and 2	Yes
CBAG4P_DMS_0002	wt ppGpp riboswitch	TECprobe-VL	Cotranscriptional	DMS	250uM ppGpp	100uM	Reps 1 and 2	Yes
CBAG4P_DMS_0003	ppGpp riboswitch, C69A	TECprobe-VL	Cotranscriptional	DMS	none	100uM	Reps 1 and 2	Yes
CBAG4P_DMS_0004	ppGpp riboswitch, C69A	TECprobe-VL	Cotranscriptional	DMS	250uM ppGpp	100uM	Reps 1 and 2	Yes
CRCBFL_BZCN_0001	wt fluoride riboswitch	TECprobe-VL	Cotranscriptional	BzCN	none	100uM	Reps 1 and 2	Yes
CRCBFL_BZCN_0002	wt fluoride riboswitch	TECprobe-VL	Cotranscriptional	BzCN	10mM NaF	100uM	Reps 1 and 2	Yes
CRCBFL_DMS_0001	wt fluoride riboswitch	TECprobe-VL	Cotranscriptional	DMS	none	100uM	Reps 1 and 2	Yes
CRCBFL_DMS_0002	wt fluoride riboswitch	TECprobe-VL	Cotranscriptional	DMS	10mM NaF	100uM	Reps 1 and 2	Yes
PFLZTP_BZCN_0001	wt ZTP riboswitch	TECprobe-VL	Cotranscriptional	BzCN	none	100uM	Reps 1 and 2	Yes
PFLZTP_BZCN_0002	wt ZTP riboswitch	TECprobe-VL	Cotranscriptional	BzCN	1mM ZMP	100uM	Reps 1 and 2	Yes
PFLZTP_DMS_0001	wt ZTP riboswitch	TECprobe-VL	Cotranscriptional	DMS	none	100uM	Reps 1 and 2	Yes
PFLZTP_DMS_0002	wt ZTP riboswitch	TECprobe-VL	Cotranscriptional	DMS	1mM ZMP	100uM	Reps 1 and 2	Yes
PFLZTPS_BZCN_0001	wt ZTP riboswitch	TECprobe-SL	Cotranscriptional	BzCN	none	100uM	No	N/A
PFLZTPS_BZCN_0002	wt ZTP riboswitch	TECprobe-SL	Cotranscriptional	BzCN	1mM ZMP	100uM	No	N/A
PFLZTPS_BZCN_0003	wt ZTP riboswitch	TECprobe-SL	Cotranscriptional	BzCN	none	100uM	No	N/A
PFLZTPS_BZCN_0004	wt ZTP riboswitch	TECprobe-SL	Cotranscriptional	BzCN	1mM ZMP	100uM	No	N/A
PFLZTPS_BZCN_0005	wt ZTP riboswitch	TECprobe-SL	Cotranscriptional	BzCN	none	500uM	No	N/A
PFLZTPS_BZCN_0006	wt ZTP riboswitch	TECprobe-SL	Cotranscriptional	BzCN	1mM ZMP	500uM	No	N/A
PFLZTPS_BZCN_0007	wt ZTP riboswitch	TECprobe-SL	Cotranscriptional	BzCN	none	500uM	No	N/A
PFLZTPS_BZCN_0008	wt ZTP riboswitch	TECprobe-SL	Cotranscriptional	BzCN	1mM ZMP	500uM	No	N/A
