# Supplementary material for: Observation of coordinated RNA folding events by systematic cotranscriptional RNA structure probing
Source: Nat Commun. 2023 Nov 29;14:7839. doi: 10.1038/s41467-023-43395-9 (PMC10687018; doi:10.1038/s41467-023-43395-9)
Supplement: Supplementary file 3 — Description of Additional Supplementary Files [file 41467_2023_43395_MOESM3_ESM.pdf]

Description of Additional Supplementary Files

File Name: Supplementary Data 1
Description: Reagent source and catalog number tables
