# Supplementary material for: Observation of coordinated RNA folding events by systematic cotranscriptional RNA structure probing
Source: Nat Commun. 2023 Nov 29;14:7839. doi: 10.1038/s41467-023-43395-9 (PMC10687018; doi:10.1038/s41467-023-43395-9)
Supplement: Supplementary file 5 — Reporting Summary [file 41467_2023_43395_MOESM5_ESM.pdf]

nature portfolio

Corresponding author(s): Eric J. Strobel

Last updated by author(s): Oct 30, 2023

Reporting Summary

Nature Portfolio wishes to improve the reproducibility of the work that we publish. This form provides structure for consistency and transparency in reporting. For further information on Nature Portfolio policies, see our Editorial Policies and the Editorial Policy Checklist.

Statistics

For all statistical analyses, confirm that the following items are present in the figure legend, table legend, main text, or Methods section.

n/a	Confirmed	
☐	☒	The exact sample size (*n*) for each experimental group/condition, given as a discrete number and unit of measurement
☐	☒	A statement on whether measurements were taken from distinct samples or whether the same sample was measured repeatedly
☒	☐	The statistical test(s) used AND whether they are one- or two-sided<br>*Only common tests should be described solely by name; describe more complex techniques in the Methods section.*
☒	☐	A description of all covariates tested
☒	☐	A description of any assumptions or corrections, such as tests of normality and adjustment for multiple comparisons
☐	☒	A full description of the statistical parameters including central tendency (e.g. means) or other basic estimates (e.g. regression coefficient) AND variation (e.g. standard deviation) or associated estimates of uncertainty (e.g. confidence intervals)
☒	☐	For null hypothesis testing, the test statistic (e.g. *F*, *t*, *r*) with confidence intervals, effect sizes, degrees of freedom and *P* value noted<br>*Give P values as exact values whenever suitable.*
☒	☐	For Bayesian analysis, information on the choice of priors and Markov chain Monte Carlo settings
☒	☐	For hierarchical and complex designs, identification of the appropriate level for tests and full reporting of outcomes
☒	☐	Estimates of effect sizes (e.g. Cohen's *d*, Pearson's *r*), indicating how they were calculated

Our web collection on statistics for biologists contains articles on many of the points above.

Software and code

Policy information about availability of computer code

Data collection	No software were used for data collection.
Data analysis	TECprobe data were processed and analyzed by fastp v0.21.0, cotrans_preprocessor v1.0.0 (this work), and ShapeMapper2 v2.1.5. RNA structure prediction was performed using the RNAstructure v6.4 Fold command in the custom script assess_fold_0.0.7. All custom software used for data processing and visualization are freely available at https://github.com/e-strobel-lab/.

For manuscripts utilizing custom algorithms or software that are central to the research but not yet described in published literature, software must be made available to editors and reviewers. We strongly encourage code deposition in a community repository (e.g. GitHub). See the Nature Portfolio guidelines for submitting code & software for further information.

Data

Policy information about availability of data

All manuscripts must include a data availability statement. This statement should provide the following information, where applicable:

- Accession codes, unique identifiers, or web links for publicly available datasets
- A description of any restrictions on data availability
- For clinical datasets or third party data, please ensure that the statement adheres to our policy

The raw sequencing data generated in this study have been deposited in the Sequencing Read Archive (https://www.ncbi.nlm.nih.gov/sra) with the BioProject accession code PRJNA929456. Individual BioSample accession codes are available in Supplementary Table 4. The processed reactivity data have been deposited in

the RNA Mapping Database (https://rmdb.stanford.edu/). Individual accession codes for each data set are available in Supplementary Table 5. The ShapeMapper2 output files for these data have been deposited in Zenodo (DOI: 10.5281/zenodo.7640593). The TIFF images of all gels generated in this study have been deposited in Zenodo (DOI: 10.5281/zenodo.10041572). Source data are provided with this paper. The crystal structures of the T. carboxydivorans ZTP, T. petrophila fluoride, T. mathranii PRPP G96A (ppGpp-binding), and Sulfobacillus acidophilus DSM 10332 riboswitches used in this study are available in the RSCB Protein Data Bank under the accession codes 4ZNP, 4ENC, 6CK4, and 6DMC. Cotranscriptional SHAPE-Seq Data for the ZTP and fluoride riboswitches used in this study was downloaded from the source data associated with the original publications.

Research involving human participants, their data, or biological material

Policy information about studies with human participants or human data. See also policy information about sex, gender (identity/presentation), and sexual orientation and race, ethnicity and racism.

Reporting on sex and gender	n/a
Reporting on race, ethnicity, or other socially relevant groupings	n/a
Population characteristics	n/a
Recruitment	n/a
Ethics oversight	n/a

Note that full information on the approval of the study protocol must also be provided in the manuscript.

Field-specific reporting

Please select the one below that is the best fit for your research. If you are not sure, read the appropriate sections before making your selection.

☒ Life sciences ☐ Behavioural & social sciences ☐ Ecological, evolutionary & environmental sciences

For a reference copy of the document with all sections, see nature.com/documents/nr-reporting-summary-flat.pdf

Life sciences study design

All studies must disclose on these points even when the disclosure is negative.

Sample size	Cotranscriptional TECprobe experiments were performed using a sample size of n=2. This sample size was chosen and is sufficient due to the reproducibility of in vitro RNA chemical probing experiments. As shown in Figure 2e-g and Supplementary Figure 1d-f, after neighboring transcript smoothing was applied, the Pearson Correlation Coefficient was >0.86 in all cases.
Data exclusions	No samples were excluded from the analyses.
Replication	All cotranscriptional TECprobe experiments are n=2 independent replicates. All replication attempts were successful. The correlation of replicate reactivity measurements for the WT ZTP, fluoride, and ppGpp riboswitches are plotted in Figure 2e-g and Supplementary Figure 1d-f. The equilibrium TECprobe-ML experiment for the ppGpp riboswitch was performed once because the observations made from the equilibrium chemical probing data agreed with the observations made for the same system in cotranscriptional TECprobe experiments.
Randomization	Randomization is not relevant to our study because our biochemical reactions are handled uniformly except for the specific condition being tested.
Blinding	Blinding is not relevant to our study because the experimental measurements are automated and deterministic, and are therefore not subject to human biases.

Reporting for specific materials, systems and methods

We require information from authors about some types of materials, experimental systems and methods used in many studies. Here, indicate whether each material, system or method listed is relevant to your study. If you are not sure if a list item applies to your research, read the appropriate section before selecting a response.

Materials & experimental systems

n/a	Involved in the study
☒	☐ Antibodies
☒	☐ Eukaryotic cell lines
☒	☐ Palaeontology and archaeology
☒	☐ Animals and other organisms
☒	☐ Clinical data
☒	☐ Dual use research of concern
☒	☐ Plants

Methods

n/a	Involved in the study
☒	☐ ChIP-seq
☒	☐ Flow cytometry
☒	☐ MRI-based neuroimaging

Plants

Seed stocks	n/a
Novel plant genotypes	n/a
Authentication	n/a
